# Comparative Analysis of Chloroplast Genomes of *Dalbergia* Species for Identification and Phylogenetic Analysis

**DOI:** 10.3390/plants11091109

**Published:** 2022-04-20

**Authors:** Hoi-Yan Wu, Kwan-Ho Wong, Bobby Lim-Ho Kong, Tin-Yan Siu, Grace Wing-Chiu But, Stacey Shun-Kei Tsang, David Tai-Wai Lau, Pang-Chui Shaw

**Affiliations:** 1 Li Dak Sum Yip Yio Chin R & D Centre for Chinese Medicine, The Chinese University of Hong Kong, Shatin, Hong Kong, China; karenwhy@cuhk.edu.hk (H.-Y.W.); konglimho@yahoo.com.hk (B.L.-H.K.); 2 Shiu-Ying Hu Herbarium, School of Life Sciences, The Chinese University of Hong Kong, Shatin, Hong Kong, China; kwanhowong@cuhk.edu.hk (K.-H.W.); joycesiuty@hotmail.com (T.-Y.S.); lautaiwai@cuhk.edu.hk (D.T.-W.L.); 3 School of Life Sciences, The Chinese University of Hong Kong, Shatin, Hong Kong, China; gracebut@link.cuhk.edu.hk (G.W.-C.B.); Stacey.tsang@link.cuhk.edu.hk (S.S.-K.T.); 4 School of Biological Sciences, The University of Hong Kong, Hong Kong, China; 5 State Key Laboratory of Research on Bioactivities and Clinical Applications of Medicinal Plants (The Chinese University of Hong Kong) and Institute of Chinese Medicine, The Chinese University of Hong Kong, Shatin, Hong Kong, China

**Keywords:** *Dalbergia*, chloroplast genomes, phylogenetic analysis, molecular authentication

## Abstract

*Dalbergia* L.f. is a pantropical genus consisting of 269 species of trees, shrubs, and woody lianas. This genus is listed in *CITES* Appendices because of illegal logging and trafficking driven by the high economic value of its heartwood. Some species are also used medicinally. Species identification of *Dalbergia* timber and herbs is challenging but essential for *CITES* implementation. Molecular methods had been developed for some timber species, mostly from Madagascar and Southeast Asia, but medicinal species in south China were usually not included in those studies. Here, we sequenced and assembled the chloroplast genomes of five *Dalbergia* species native to Hong Kong, four of which are medicinal plants. Our aim is to find potential genetic markers for the identification of medicinal *Dalbergia* species based on divergence hotspots detected in chloroplast genomes after comparative and phylogenetic analysis. *Dalbergia* chloroplast genomes displayed the typical quadripartite structure, with the 50 kb inversion found in most Papilionoideae lineages. Their sizes and gene content are well conserved. Phylogenetic tree of *Dalbergia* chloroplast genomes showed an overall topology similar to that of *ITS* sequences. Four divergence hotspots (*trnL*(UAA)-*trnT*(UGU), *ndhG-ndhI*, *ycf1a* and *ycf1b*) were identified and candidate markers for identification of several *Dalbergia* species were suggested.

## 1. Introduction

*Dalbergia* L.f. is a pantropical genus with 269 accepted species of trees, shrubs, and woody lianas according to Plants of the World Online [1] and the Legume Data Portal [2] of the Legume Phylogeny Working Group. The genus is native to more than 130 countries [1] in the tropical and subtropical zones, mainly in Asia, Africa, and Central and South America [3]. It belongs to tribe Dalbergieae, subfamily Papilionoideae of family Fabaceae [4]. Members of the genus *Dalbergia* is of high economic value. The heartwood of some of the *Dalbergia* species are known as rosewood, or Hongmu in Chinese. Because of its distinctive color and durability, rosewood is a precious timber for making high-end furniture, valuable carvings, and musical instruments. Fifteen *Dalbergia* species have been listed as authentic sources of rosewood in *National Standard for Hongmu* (GB/T 18107-2017) by the Standardization Administration of China [5]. The high value of rosewood timber has driven illegal logging, threatening not just the timber species, but the forest habitat and all species living there. To regulate the international trade of *Dalbergia* timber, the whole genus *Dalbergia* was included in the *Convention on International Trade in Endangered Species of Wild Fauna and Flora* (*CITES*) Appendix II in 2016, except for *Dalbergia nigra* (Brazilian rosewood) which was already listed in Appendix I.

Besides, some *Dalbergia* species are medicinal plants. According to the Medicinal Plant Names Services provided by Royal Botanic Gardens, Kew [6], 57 *Dalbergia* species have been cited in medicinal sources, i.e. pharmacopoeias and reference collections, all over the world. Heartwood of *Dalbergia odorifera* is a Chinese materia medica listed in the *Chinese Pharmacopoeia* [7]. It has been used traditionally to treat cardiovascular diseases and blood disorders for its qi- and blood-invigorating effect. It can also be used to stop bleeding and relieve pain [7,8]. In addition to *D. odorifera, D. assamica, D. benthamii, D. hainanensis, D, hancei, D. hupeana, D. millettii, D. mimosoides, D. obtusifolia, D. rimosa, D. sissoo*, and *D. yunnanensis* have all been listed as sources of medicinal materials in *Zhonghuabencao*, a reference collection of Chinese medicinal herbs [9]. Except for *D. odorifera* (listed in the *Chinese Pharmacopoeia*) and *D. hancei* (listed in the *Standards of Zhuang Materia Medica of Guangxi Zhuang Autonomous Region*), these medicinal materials have not been listed in any official standards in China. There is no official guidance for morphological identification and no validated methods for quality control of these herbal materials.

It is also not easy to distinguish between different timber species, especially in the form of finished products. A reliable identification method must be in place to support law enforcement. Traditional wood identification relies on wood anatomy, based on both macroscopic and microscopic characteristics. Online databases and recognition tools, such as the InsideWood database [10] and CITESwoodID [11,12], have been developed and made publicly available. However, customs inspectors usually do not have sufficient training, nor do they have access to laboratory equipment and reference collections for microscopic scrutiny [11]. These databases and tools, expectedly, only cover trees, but not shrubs and lianas of *Dalbergia*. Various molecular techniques have been employed for the identification of *Dalbergia* species, such as random amplified polymorphic DNAs [13], simple sequence repeats [14], and DNA barcoding [15,16,17,18,19,20]. These recent studies on DNA barcoding concluded that multi-loci combinations have better discriminatory power, but they suggested different multi-loci combinations, such as *rbcL*+*matK*+*ITS* [16,17], *ITS2*+*trnH*-*psbA* [18,19], and *rbcL*+*matK*+*trnL* [20], based on different *Dalbergia* species involved in the analyses.

*Dalbergia* L.f. is a genus with pantropical distribution whose complete infrageneric classification is difficult [15]. The first attempt was made by George Bentham in *A Synopsis of the Dalbergieae, a tribe of the Leguminosae* in 1860 [21]. Based on geographical distribution and morphological evidence, he classified 64 species under six series and several subdivisions. David Prain contributed to the infrageneric classification of Asian *Dalbergia* in *The species of Dalbergia of southeastern Asia* [22]. He classified 86 species under two subgenera (*Sissoa* and *Amerimnon*), five sections (*Triptolemea*, *Podiopetalum*, *Endespermum*, *Miscolobium*, and *Dalbergaria*) and 24 groups, based on floral morphologies. In 1989, André de Carvalho published his treatment on Brazilian *Dalbergia* [23,24]. He classified 39 species with two varieties under five sections (*Dalbergia*, *Triptolemae*, *Selenolobium*, *Pseudecastaphyllum*, and *Ecastaohyllum*) based on the morphological evidence of reproductive organs [23]. However, molecular phylogeny based on *ITS* sequences do not match these treatments completely [15]. Hence, more molecular data are needed to facilitate the infrageneric classification of *Dalbergia*.

Complete chloroplast (cp) genomes have been proposed to be super-barcodes that provide higher discriminatory power than conventional barcodes [25]. By comparing complete chloroplast genomes of different species systematically, genetic hotspots with potential for species identification could be detected and phylogenetic relationship among studied species could be revealed. Such studies have been carried out on various plant groups, such as feather grasses (*Stipa* species) [26], *Ilex* species [27], *Fritillaria* species [28], and *Hedyotis* species [29]. In 2019, Song et al. assembled the cp genomes of nine *Dalbergia* species and identified eight divergence hotspots after comparing the nine sequences [30]. Chloroplast genomes of more *Dalbergia* species have been published, but those of medicinally used *Dalbergia* species in South China are still unavailable. In this study, we have assembled the complete chloroplast genomes of five *Dalbergia* species native to Hong Kong. All but one species, *D. candenatensis*, are medicinal plants. A total of 46 complete cp genomes from the 26 *Dalbergia* species available, including all but one medicinally used *Dalbergia* species in China, have been analyzed for divergence hotspots identification and study of phylogenetic relationship. To check if the divergence hotspots are also present in the mitochondrial genome, which may hamper the use of those hotspots for identification, the newly assembled cp genomes were compared against the complete mitochondrial genome of *D. odorifera* by BLAST to identify potential mitochondrial plastid DNAs. Finally, the divergence hotspots and internal transcribed spacer 2 (*ITS2*), a popular DNA barcode, were evaluated for their potential for species discrimination.

## 2. Results

### 2.1. Genome Sequencing and Features of Assembled Chloroplast Genomes

**Figure 1:**
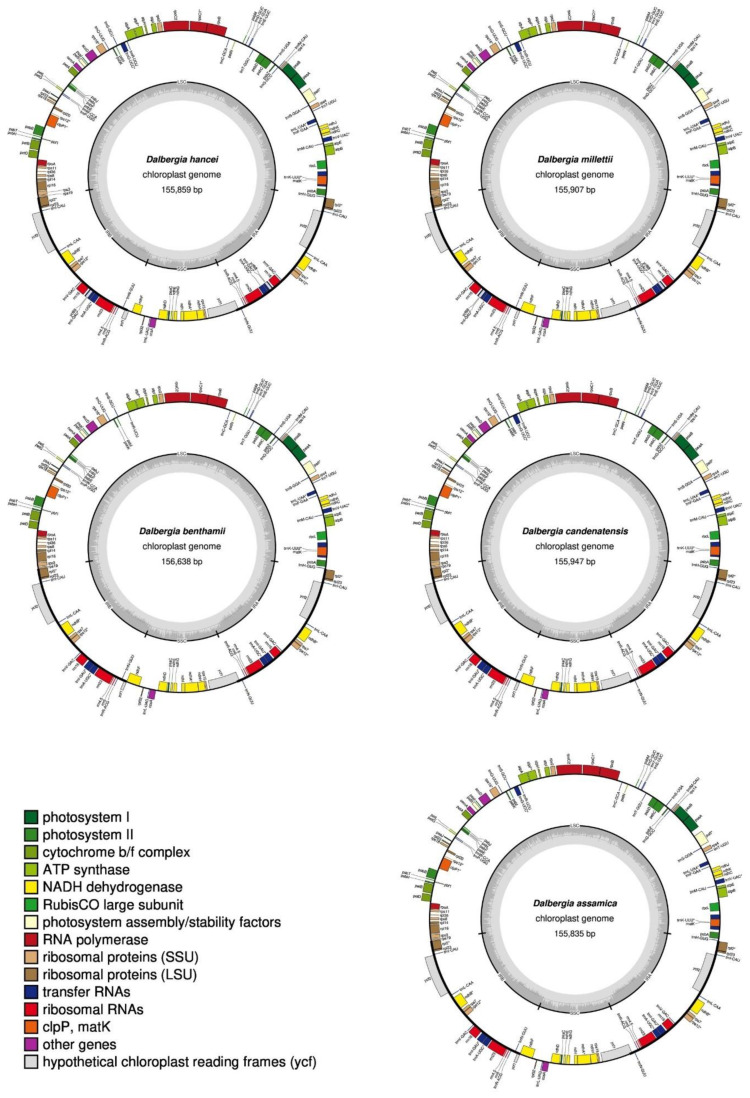
Genome maps of the five *Dalbergia* chloroplast genomes.

The Illumina NovaSeq 6000 system produced 16,579,278 to 19,675,290 clean, paired-end reads per species. Complete cp genomes were assembled by de novo assembly. Clean, paired-end reads were mapped to the assembled contigs for validation. The cp genomes obtained had mean coverage ranging from 154.29 X to 829.73 X (Table S1). Figure 1 shows the cp genome maps of all five *Dalbergia* species and characteristics of the cp genomes are listed in Table 1. Among them, the cp genome of *D. benthamii* is the largest in size at 156,638 bp. The smallest genome belongs to *D. assamica*, with a genome size of 155,835 bp. All cp genomes demonstrated the quadripartite structure typical of angiosperms, with a large single copy (LSC) ranging from 85,253 bp to 85,767 bp, inverted repeats (IRs) between 25,671 bp and 25,742 bp in size, and a small single copy (SSC) ranging from 18,978 bp to 19,427 bp. The GC content is between 36.02% and 36.19%, similar to the value previously reported [30].

When only single copies of duplicated genes are counted, the total number of genes in the assembled *Dalbergia* cp genomes ranged from 111 to 114, with 75 protein-coding genes, 2–3 hypothetical proteins, 30–32 tRNAs, and 4 rRNAs. All the genes were categorized according to the gene functions and shown in Table 2. There are two genes that are present only in some of the *Dalbergia* species studied. The *trnG*-UCC gene is absent in the genome of *D. benthamii* but is present in the other four genomes. The *ycf68* gene is only present in *D. hancei* and *D. millettii*. The number of duplicated genes in the IR regions ranged from 18 to 19, depending on the presence/absence of *ycf68*. Among the duplicated genes, there are seven tRNA genes, four rRNA genes, five protein-coding genes and two to three hypothetical genes (*ycf1*, *ycf2* and *ycf68*). Sixteen genes that harbor intron(s) were found, with 13 genes carrying one intron and three genes, *pafI*, *rps12*, and *clpP1*, containing two introns.

### 2.2. Sequence Repeat Elements

**Figure 2:**
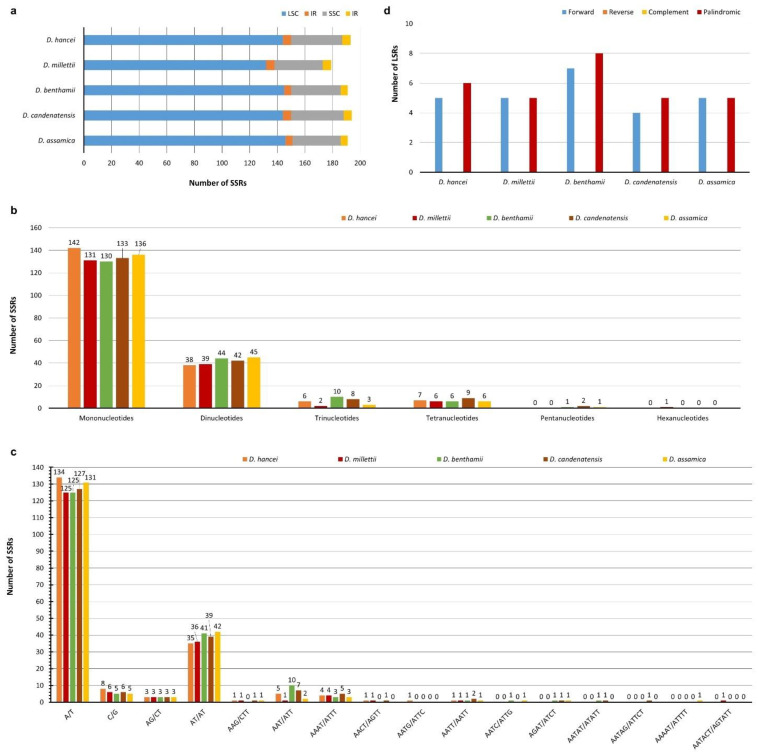
Analysis of repeats in the five *Dalbergia* chloroplast genomes. (**a**) Number of SSRs in different regions of *Dalbergia* cp genomes. (**b**) Frequency of SSRs classified by the types of repeats. (**c**) Frequency of different SSR motifs. (**d**) Number of different types of long sequence repeats.

A total of 179–194 SSRs were identified in the five assembled *Dalbergia* cp genomes (Figure 2a). *D. millettii* has the smallest number of SSRs, while the rest have 191 or more. The LSC region has the highest number of SSRs ranging from 132 to 146 (73.74–76.44%), followed by the SSC region with 35–38 SSRs (18.32–19.59%) and the IR region with 5–6 SSRs (2.62–3.35%). However, if we take the length of each region into account, the SSC region has a higher SSR density (number of SSRs per nucleotide) (0.001824–0.001978) than LSC (0.001546–0.001712) (Table S2). Mononucleotide is the most common type of SSRs (130–142, average percentage 70.89%), followed by dinucleotide (38–45, average percentage 21.94%), trinucleotide (2–10, average percentage 3.06%), and tetranucleotide (6–9, average percentage 3.59%) (Figure 2b). Pentanucleotide is rare (1–2) and was only found in *D. benthamii*, *D. candenatensis*, and *D. assamica*. Only one hexanucleotide (AATACT/AGTATT) was detected exclusively *in D. millettii*. Same as other *Dalbergia* cp genomes previously reported [24], SSRs of these five *Dalbergia* cp genomes are AT-rich. There are 125–134 A/T mononucleotide repeats and 35–42 AT/AT dinucleotide repeats but only 5–8 mononucleotide C/G repeats and 3 AG/CT dinucleotide repeats (Figure 2c). An AATG/ATTC tetranucleotide was only found in *D. hancei*. AATAG/ATTCT and AAAAT/ATTTT pentanucleotides were only detected in *D. candenatensis* and *D. assamica*, respectively. Complex repeat regions were analyzed with the REPuter algorithm (Figure 2d). Among the five chloroplast genomes, there are only 9–15 LSRs, with 4–7 forward repeats and 5–8 palindromic repeats. Reverse repeats and complement repeats were not detected. The largest repeat is a 287 bp sequence. There were two copies of this large repeat, one between the *rpl23* gene and *trnI*-CAU gene and one in the *ycf2* gene, in each IR region in all studied species. This repeat element is also present in other legumes, such as *Glycine max*, *Lotus japonicus* [31], *Phaseolus vulgaris* [32], and *Lupinus luteus* [33].

### 2.3. Comparative Genome Analysis

**Figure 3:**
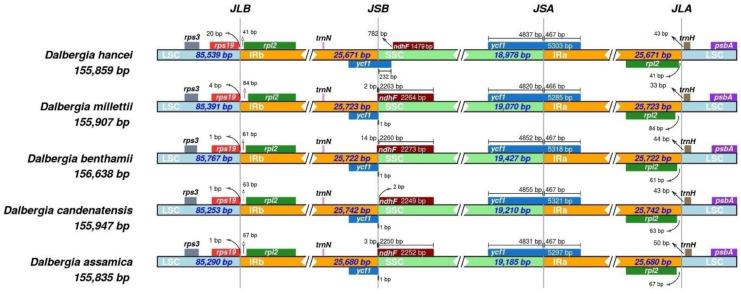
Comparison of the junction regions of the cp genomes of the five *Dalbergia* species. (JLB: junction between LSC and IRB; JSB: junction between SSC and IRB; JSA: junction between SSC and IRA; JLA: junction between LSC and IRA).

Looking into the gene arrangement and border regions, it was found that the gene arrangement of the cp genomes of the five *Dalbergia* species is quite conserved (Figure 3 and Figure S1). There is no gene spanning the junction of LSC/IRb and IRa/LSC. The *rps19* gene is 1–20 bp in front of and the *rpl2* gene is 41–84 bp behind the junction of LSC/IRb, while the *rpl2* gene and *trnH* gene are flanking the IRa/LCS junction. At the junction of IRb/SSC, the *ycf1* pseudogene spanned across the boundary. For *D. hancei*, the *ycf1* pseudogene is longer in length (699 bp), with 232 bp of the gene located in the SSC region. For the other four species, their *ycf1* pseudogene is shorter in length (467–468 bp) and the first nucleotide of the SSC region is the last base of the stop codon. The *ndhF* gene is located in the SSC region of the junction. The *ndhF* gene of *D. hancei* is truncated with a length of only 1479 bp and it is 782 bp away from the junction of IRb/SSC. For the other four *Dalbergia* species, their *ndhF* gene is intact with a length of 2249–2273 bp. They are either just spanning across the junction, with 2–14 bp of the gene at the IRb region (*D. millettii*, *D. benthamii*, and *D. assamica*), or a mere 2 bp away from the border at the SSC region (*D. candenatensis*). The full-length *ycf1* gene spans across the SSC/IRa junction in all five species.

**Figure 4:**
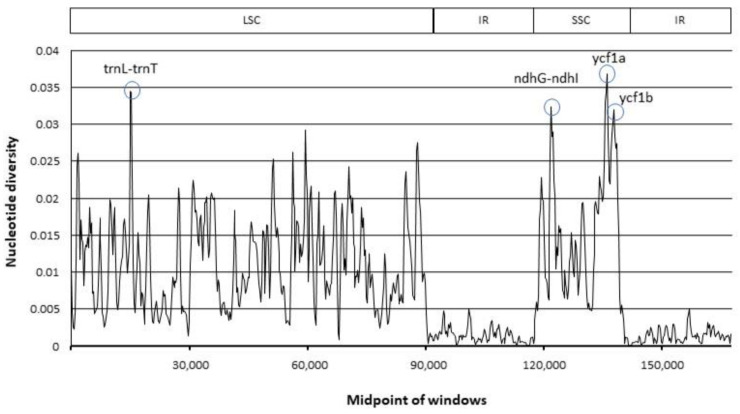
Sliding window analysis of 46 *Dalbergia* cp genomes.

In the sliding window analysis, four regions with nucleotide diversity values (*p* ≥ 0.03) were identified as divergence hotspots (Figure 4). The most variable hotspot is *ycf1a*, (*p* = 0.037), followed by the *trnL*-*trnT* intergenic spacer (*p* = 0.035), *ndhG*-*ndhI* intergenic spacer (*p* = 0.032), and *ycf1b* (*p* = 0.032).

### 2.4. Detection of Potential MTPTs

Inter-organelle DNA transfer and mitochondrial plastid DNAs (MTPTs) were first reported in maize in 1982 [34,35]. Since then, different studies have shown the prevalence of MTPTs in angiosperms [36,37,38]. Recently, it was reported that *matK* and *rpoB*, two universal plastid barcode regions, were identified as MTPTs in two *Cynanchum* species. Some of the MTPTs could be co-amplified with plastid barcoding markers, potentially confounding a molecular authentication experiment based on plastid sequences [39]. In order to check if the divergence hotspots identified are present as MTPTs, BLASTn was performed to compare the cp genome sequences with the published complete mitochondrial genome sequence of *D. odorifera*, the only mitogenome of *Dalbergia* available. Summarized results are listed in Table 3. Detailed information can be found in Table S3. There were 44–45 regions in the cp genomes of the five *Dalbergia* species, as well as the reference sequence of *D.* *odorifera* (NC_049008.1), identified as MTPTs, accounting for 9.98–10.51% of the cp genomes. More than half of the MTPTs (28–33) belonged to transfer RNA and ribosomal RNA. The rest were protein-coding MTPTs (11–12) and non-coding MTPTs from intergenic spacer regions (2–5 only). The majority of the MTPTs (30–32) are from the IR regions, probably because of its prevalence in ribosomal RNA genes. The protein-coding MTPTs are from *ndhB*, *ycf1*, *ycf2*, *atpA*, *atpB*, *aptE*, and *rps12*. The MTPTs containing the *ndhB* gene are the longest, with a length of 2493 bp. The four divergence hotspots have not been identified as MTPTs.

### 2.5. Phylogenetic Analysis

**Figure 5:**
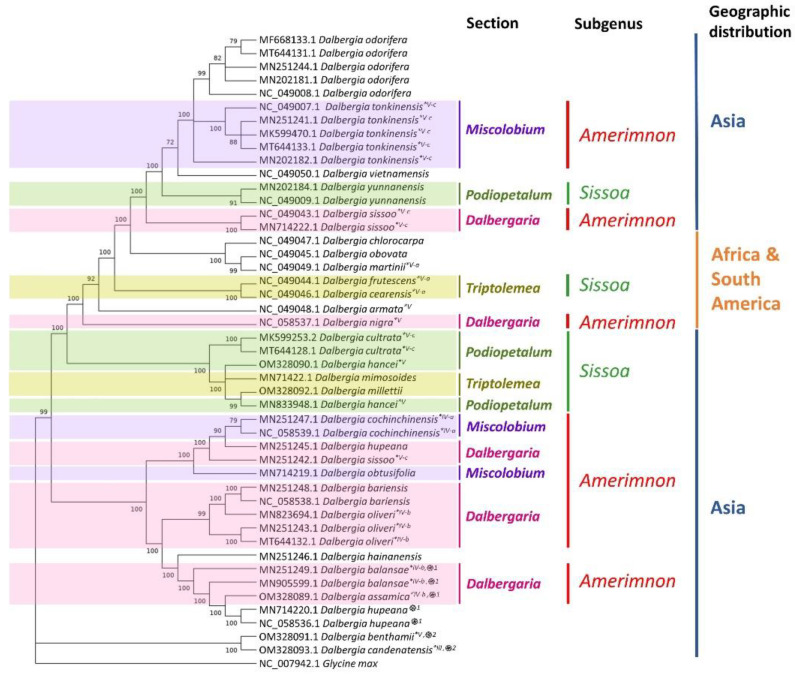
Maximum likelihood tree of 46 chloroplast genomes of 26 *Dalbergia* species, with *Glycine max* as an outgroup. Species included in the phylogenetic study based on *ITS* sequences by Vatanparast et al. [15] were marked with an asterisk, followed by the clade number assigned in that study. ֍1: Extension of *ycf68* gene. ֍2: Loss of *ycf68* gene because of internal stop codon.

The five newly assembled cp genomes were analyzed together with all *Dalbergia* cp genomes available on NCBI GenBank, except for the few dubious ones stated in Methods. The condensed maximum likelihood tree is shown in Figure 5. Original ML tree with genetic distances is shown in Figure S2. *D. hancei* and *D. millettii* obtained in this study are in the same clade as *D. mimosoides* and *D. hancei*. *D. assamica* was grouped into a small paraphyletic clade together with *D. hypeana*, *D. balansae*, and *D. hainanensis*, which is in line with previous studies using *ITS* sequences [15] and *rbcL*+*matK*+*ITS* sequences [16]. *D. benthamii* and *D. candenatensis* were grouped into a small clade distinct from all other *Dalbergia* species. This is in agreement with the study of Hartvig et al., but different from the study using *ITS* sequences [15], in which *D. benthamii* and *D. candenatensis* were in different clades, Clade V and Clade III.

### 2.6. Candidate Markers for Identification of Medicinal Dalbergia Species

As there are only 1–5 cp genomes for each of the 26 *Dalbergia* species analyzed, the intraspecific and interspecific variations of the *Dalbergia* species cannot be fully represented. We could only attempt to evaluate whether the hotspots could give monophyletic clades for species of interest and would be worthy of further investigations. Neighbor-joining trees were built for the four divergence hotspots (Figure S3). All four hotspots gave monophyletic clades for *D. cultrata* and *D. bariensis*. The *trnL*(UAA)-*trnT*(UGU) and *ycf1b* gave monophyletic clades for *D. cochinchinensis*. In addition, the *trnL*(UAA)-*trnT*(UGU) formed monophyletic clade for *D. hancei*, while the *ndhG*-*ndhI* intergenic spacer gave monophyletic clades for *D. yunnanensis* and *D. hancei*. The *ycf1b* is the only hotspot that could produce monophyletic clade for *D. odorifera*. Since this evaluation is not applicable to species with only one cp genome analyzed, we also looked into the discriminatory power of *ITS2* sequences using *ITS* sequences with voucher specimen number in GenBank. In Figure S4, monophyletic clades could be obtained for *D. retusa*, *D. sissoo*, *D. trichocarpa*, *D. hancei*, *D. millettii*, *D. dyeriana*, *D. melanoxylon*, *D. cochinchinensis*, *D. nigra*, *D. obtusifolia*, *D. miscolobium*, *D. candenatensis*, *D. pinnata*, *D. nigrescens*, and *D. stevensonii*. These regions could be potential candidate markers for identifying the corresponding species.

## 3. Discussion

Extensive structural rearrangements, gene loss and mutations have occurred in chloroplast genomes of different subfamilies of Fabaceae, which is therefore regarded as a “model system for understanding chloroplast genome evolution” [40]. Same as other *Dalbergia* cp genomes available in GenBank, the five newly assembled cp genomes displayed the quadripartite structure typical of angiosperms, as well as the 50 kb inversion commonly found in most taxa of Papilionoideae [41]. In cp genomes of core Genistoid species, an inversion of ~36 kb is present. The 36 kb inversion was believed to be caused by flip-flop recombination of a 29 bp repeat near the 3′ end of two *trnS* genes (*trnS*-GCU and *trnS*-GGA) [33,42]. *Dalbergia* cp genomes do not possess the ~36 kb inversion specific to core Genistoid, which is embedded within the 50 kb inversion. In the cp genomes of the *Dalbergia* species, and those of other species in Dalbergieae tribe (based on all Dalbergieae cp genomes available in GenBank as of 15 March 2022), there was a base substitution in the 29 bp fragment of *trnS*-GCU. This might have prevented the inversion from happening. All *Dalbergia* cp genomes have lost the *infA* gene and *rpl22* gene, which is common in all legumes [43]. The *rps16* gene, which has been reported to be lost in different legume taxa [40,42,44], was present in all *Dalbergia* cp genomes, except for *Dalbergia nigra* because of a unique InDel (Figure S5). The *ycf68* gene was annotated in only two of the five newly assembled *Dalbergia* cp genomes and 16 out of 62 *Dalbergia* cp genomes available in GenBank (as of 16 March 2022). However, when we extracted the exon sequences of *ycf68* from the alignment for translation, we found that most *Dalbergia* species have the same amino acid sequences for this gene (Figure S6). There is an InDel that caused an extension of the *ycf68* gene in five cp genomes of *D. balansae*, *D. assamica*, and *D. hupeana*, which were clustered together in the phylogenetic tree (Figure 5). The *ycf68* is a cryptic reading frame located within the intron of *trnI*-GAU. It was first identified in *Oryza sativa*, annotated as ORF133 (GenBank accession X15901.1) [45,46]. However, because of the prevalence of internal stop codon in amino acid sequences, ambiguous codon usage across different taxa, and the lack of sequence conservation beyond the normal level for non-coding regions of the IR region, it has been suggested that *ycf68* is not a protein-coding gene [47].

The overall topology of the phylogenetic tree of *Dalbergia* cp genomes is generally similar to the one obtained with *ITS* sequences [15]. It is first divided into two clades. The small clade contains *D. candenatensis*, which was in Clade III in the study with *ITS* sequence. The large clade contains two big subclades, corresponding to Clade IV and Clade V assigned by Vatanparast et al. The major discrepancy is on *D. benthamii*, which was grouped into the small clade with *D. candenatensis* in our study but assigned to Clade V by Vatanparast et al. In another study using *rbcL*+*matK*+*ITS* [16], however, *D. benthamii* and *D. candenatensis* were also in one small subclade, which was also sister to a large subclade containing most *Dalbergia* species in their study. It was also noticed that the two cp genomes of *D. hancei* were not clustered together. One was clustered with *D. millettii* (OM328092) and together they were sister to *D. mimosoides* (MN714221) and the other cp genome of *D. hancei* (OM328090). Our results showed the close phylogenetic relationship between *D. hancei* and *D. millettii*, which was also supported by the phylogenetic tree of *ITS2* sequences (Figure S4), in which *D. hancei* and *D. milletti* were distinct sister taxa further clustered together. Pairwise distance analysis showed that the genetic distance between the two cp genomes of *D. hancei* was 0.0031, which was smaller than the within group mean distance of *D. sissoo* (0.0096) and *D. oliveri* (0.0036) (Table S4). This showed that the difference between the two cp genomes of *D. hancei* was smaller than the intraspecific differences of *D. sissoo* and *D. oliveri*. Although there have been several studies on DNA barcoding and phylogenetic analysis of *Dalbergia*, few of them included sequences of *D. hancei*. Vatanparast et al. clustered *D. hancei* in Clade V based on ITS sequences [15]. Using *rbcL*+*matK*+*ITS* [16], Hartvig et al. also reported that their two samples of *D. hancei* were not in the same cluster. One of their *D. hancei* samples was clustered with *D. mimosoides*, *D. dyeriana*, and *D. cultrata*, similar to the results of Vatanparast et al. [15] and of this study. However, their other sample was clustered with *D. oliveri* and *D. cana* in a completely different clade. More samples of *D. hancei* would be needed to further elucidate its intraspecific distance and its relationship with *D. millettii. Dalbergia* species included in this study have been grouped into two subgenera and multiple sections according to Prain [22] and de Carvalho [23]. Our results show that these sections are non-monophyletic. This is not surprising as the subdivision of some sections was not natural. For instance, the section *Triptolemeais* cannot be naturally separated from the section *Podiopetalum*. In 1904, Prain [22] commented that the two sections “pass into each other at various points” and they cannot be easily distinguished “because every individual character breaks down”.

In this study, we identified four divergence hotspots among *Dalbergia* cp genomes. These four hotspots had also been detected in the previous study, which identified eight hotspots in total [30]. The reduction in the number of divergence hotspots could be because of the increase in number of cp genomes, including newly analyzed species and multiple cp genomes of the same species whenever possible. This would allow us to identify divergence hotspots that can distinguish more *Dalbergia* species. While the entire genus *Dalbergia* is listed in *CITES* Appendices, we are particularly interested in medicinal *Dalbergia* species, such as *D. odorifera* (listed in the *Chinese Pharmacopoeia*) and *D. hancei* (listed in the *Standards of Zhuang Materia Medica of Guangxi Zhuang Autonomous Region*), as well as *D. assamica* and *D. millettii*, which are used as folk medicine in South China. Our results suggest several candidate markers, *ycf1b* for *D. odorifera*, *trnL*(UAA)-*trnT*(UGU), *ndhG*-*ndhI* and *ITS2* for *D. hancei*, and *ITS2* for *D. millettii*. For *D. nigra*, the only *Dalbergia* species listed in *CITES* Appendix I, *ITS2* appears to be a good candidate. Unfortunately, the sequence data currently available is still insufficient for thorough evaluation of the discriminatory power of candidate markers. For one thing, GenBank has currently only collected cp genomes of 26 *Dalbergia* species. For barcode sequences that are more abundant in GenBank, there are only 96 *Dalbergia* species with *ITS* sequences available and voucher specimen numbers deposited. Even for *D. nigra*, there are only four *ITS* sequences provided by two research groups. *Dalbergia* is a genus containing 269 species that are widespread and require protection. Concerted effort from the scientific community is needed to enrich the genomic data of this genus. Further experiments are still needed for the generation and validation of short genetic markers (~100–200 bp) for developing an identification method applicable to timber samples and processed medicinal materials.

## 4. Material and Methods

### 4.1. Ethics Statement

Specimens of *Dalbergia assamica* and *Dalbergia candenatensis* were collected with the assistance of the Hong Kong Herbarium, Agriculture, Fisheries, and Conservation Department of the Government of the Hong Kong Special Administrative Region. Specimens of *Dalbergia benthamii*, *Dalbergia hancei*, and *Dalbergia millettii* were collected by members of the Shiu-Ying Hu Herbarium with a field collection permit in Hong Kong in 2019–2020. All collections are permitted and legal in Hong Kong.

### 4.2. Plant Material and DNA Extraction

Plant samples were identified by Dr. David Tai-Wai Lau, curator of the Shiu-Ying Hu Herbarium of the Chinese University of Hong Kong. Detailed information is listed in Table 4. Leaves were dried with silica gel and further stored in a −80 °C freezer before DNA extraction. Voucher specimens were deposited in the Shiu-Ying Hu Herbarium (Herbarium code: CUHK).

Total genomic DNA was extracted from 0.1 g dried leaves according to the spin column DNA extraction protocol for plant-derived Chinese materia medica (Annex A2 of GCMTI RD-5:2020) of the Government Chinese Medicines Testing Institute, Hong Kong SAR [48]. Quality and quantity of DNA extracts were assessed with NanoDrop Lite Spectrophotometer (Thermo Fisher Scientific, Waltham, MA, USA) and by 1% agarose gel electrophoresis, respectively.

### 4.3. Chloroplast Genome Sequencing, Assembly and Annotation

Paired-end libraries with 150 bp insert were constructed from total genomic DNA. Sequencing was performed by Novogene Bioinformatic Technology Co. Ltd. (Beijing, China) (http://en.novogene.com/, accessed on 23 March 2022) on the NovaSeq 6000 platform (Illumina Inc. San Diego, CA, USA). Approximately 3.0 Gb of raw data were generated.

CLC Assembly Cell package v5.1.1 (CLC Inc., Denmark) was used to perform quality trimming, mapping, and de novo assembly. Poor-quality reads with a Phred score below 33 were removed to obtain clean filtered reads, which were assembled into contigs with a CLC assembler CLC Assembly Cell package. Gaps were filled using GapCloser in SOAPdenovo v3.23 and contigs were re-ordered by NUCmer 3.0. Contigs were then mapped to the reference genome, *Dalbergia odorifera* (NC_049008.1). Mapped contigs were selected and assembled into complete cp genomes. Gaps between the contigs, if any, were amplified and sequenced using specific primers. Newly obtained chloroplast genomes were annotated on the GeSeq platform (https://chlorobox.mpimp-golm.mpg.de/geseq.html, accessed on 23 March 2022) [49] with manual adjustment of the start and stop codons of a few protein-coding genes, using the complete cp genomes of *D. odorifera* (NC_049008.1) and *D. martinii* (NC_049049.1) as reference. OrganellarGenomeDRAW (OGDRAW, https://chlorobox.mpimp-golm.mpg.de/OGDraw.html, accessed on 23 March 2022) [50] was used to visualize the circular genomic map of the assembled sequences. Assembled and annotated cp genome sequences were submitted to GenBank, with accession numbers listed in Table 1.

### 4.4. Repeat Sequence Analysis

MIcroSAtellite identification tools, MISA (https://webblast.ipk-gatersleben.de/misa/index.php?action=1, accessed on 23 March 2022) [51] and REPuter (https://bibiserv.cebitec.uni-bielefeld.de/reputer, accessed on 23 March 2022) [52] were used to identify simple sequence repeats (SSRs) and long sequence repeats (LSRs), respectively. SSRs with minimum numbers of repetitions of 10, 5, 4, 3, 3, 3 for mono-, di-, tri-, tetra-, penta-, and hexa-nucleotides were detected. LSRs, forward, reverse, complement, and palindromic sequences included, were detected, with a maximum computed repeat size of 50 bp and minimal repeat size of 30 bp.

### 4.5. Comparative Genome Analysis

Visualization of genome structure of newly obtained cp genomes was performed with mVISTA software (https://genome.lbl.gov/vista/mvista/submit.shtml, accessed on 23 March 2022) [53]. The Shuffle-LAGAN alignment program was chosen [54] and the cp genome of *D. hancei* (OM328090) was selected as reference. For better visualization of junction sites of the boundary regions, the online program IRscope (https://irscope.shinyapps.io/irapp/, accessed on 23 March 2022) was adopted [55]. The diagram and results obtained were manually verified and edited.

To identify divergence hotspots, all cp genome sequences available in GenBank (as of 4 January 2022) were downloaded. Accessions selected as reference sequences of GenBank were removed to avoid duplication while the reference sequences were retained. Accessions NC_036961.1 of *D. hainanensis* was also deleted because of suspected misidentification [56]. The remaining sequences were aligned with MAFFT version 7 (https://mafft.cbrc.jp/alignment/server/, accessed on 23 March 2022) [57]. Two sequences, *D. odorifera* (MT009405.1) and *D. oliveri* (NC_053827.1), were further removed as they are apparently different from other sequences of the same species. Sliding window analysis was performed using DnaSP v6.12.03 software [58]. The window length and step size were set to 600 bp and 200 bp, respectively.

### 4.6. Identification Potential Mitochondrial Plastid DNAs (MTPTs)

Potential MTPTs, mitochondrial sequences of plastid origin arising from horizontal inter-organelle DNA transfer, were identified by comparing the newly assembled cp genome sequences and the NCBI refseq of *D. odorifera* (NC_049008.1) with the mitochondrial genome sequence of *D. odorifera* (NW441235.1) using BLASTn [59] with the following parameters: an expectation value (E value) of 1e-5; a word size of 7; match/mismatch scores at 2 and -3, respectively; and gap penalty values of 5 (existence) and 2 (extension). BLAST hits with aligned length less than 50 nucleotides and a sequence identity lower than 70% were filtered. The genes that the matched regions belong to, in both chloroplast and mitochondrial genomes, were manually checked.

### 4.7. Phylogenetic Analysis

To infer the phylogenetic relationships of the *Dalbergia* species, a maximum likelihood (ML) tree was constructed based on the complete cp genomes using MEGA X software [60]. The general-time nucleotide substitution reversible model was selected. Complete deletion was chosen for handling gaps/missing data. Bootstrap replicates were set to 1000. *Glycine max* (NC_007942.1) was added as an outgroup. Pairwise genetic distance was also computed with MEGA X [60].

### 4.8. Evaluation of Divergence Hotspots and ITS2 Sequence

Sequences of the four divergence hotspots were extracted from the alignment of cp genomes to construct their own neighbor-joining (NJ) trees using MEGA X software [60] based on k2p distances. Partial deletion with 95% cutoff was chosen. Bootstrap replicates were set to 1000. *Pterocarpus indicus* (MT249115.1) was added as the outgroup. If the divergence hotspot could produce an NJ tree with a monophyletic clade containing all analyzed sequences of a certain species, we would regard the divergence hotspot as a potential marker for distinguishing that species from other congeneric species.

To obtain the sequences of internal transcribed spacer 2 (*ITS2*) for evaluation, the assembled contigs were mapped to an *ITS* sequence of *D. odorifera* (KY489987.1). We then re-mapped the clean filtered reads to the contig obtained to ensure the coverage. The *ITS* sequences obtained were then aligned with *ITS* sequences downloaded from GenBank for building a neighbor-joining tree. Only accessions annotated with a voucher number were downloaded.

## 5. Conclusions

In this study, we have sequenced and assembled the chloroplast genomes of five *Dalbergia* species native to Hong Kong. The chloroplast genomes displayed the typical quadripartite structure and the 50 kb inversion common for most Papilionoideae lineages. Size and gene content among all *Dalbergia* chloroplast genomes are quite conserved, and an abundance of SSRs have been observed. Four divergence hotspots were identified from a phylogenetic analysis containing 46 chloroplast genomes of 26 *Dalbergia* species. Candidate markers for identification of several medicinally used *Dalbergia* species were suggested based on phylogenetic trees of individual divergence hotspots and *ITS2* sequences. Our work provides the foundation to further enrich the DNA barcode and genomic data of this genus, as well as for the development and validation of short genetic markers for use in timber samples.

## Figures and Tables

**Table 1:** Summary of the five *Dalbergia* chloroplast genomes.

Species	GenBank Accession	Genome Size (bp)	LSC (bp)	IR (bp)	SSC (bp)	Total Gene Number	Protein Coding	Hypothetical Protein	tRNA	rRNA	GC%	A%	C%	G%	T%
*Dalbergia hancei*	OM328090	155,859	85,539	25,671	18,978	114	75	3	32	4	36.18	31.90	17.95	18.23	31.92
*Dalbergia millettii*	OM328092	155,907	85,391	25,723	19,070	112	75	3	30	4	36.14	31.94	17.93	18.21	31.92
*Dalbergia benthamii*	OM328091	156,638	85,767	25,722	19,427	112	75	2	31	4	36.02	31.99	17.88	18.14	31.99
*Dalbergia candenatensis*	OM328093	155,947	85,253	25,742	19,210	111	75	2	30	4	36.07	31.96	17.89	18.17	31.97
*Dalbergia assamica*	OM328089	155,835	85,290	25,680	19,185	111	75	2	30	4	36.19	31.92	17.96	18.23	31.89

**Table 2:** Genes annotated in the five *Dalbergia* chloroplast genomes.

Gene Category	Gene Function	Gene Name
Photosynthesis-related genes	Rubisco	*rbcL*
	Photosystem I	*psaA*, *psaB*, *psaC*, *psaI*, *psaJ*
	Assembly/stability of photosystem I	pafI **, *pafII*, *pbf1*
	Photosystem II	*psbA*, *psbB*, *psbC*, *psbD*, *psbE*, *psbF*, *psbH*, *psbI*, *psbJ*, *psbK*, *psbL*, *psbM*, *psbT*, *psbZ*
	ATP synthase	*atpA*, *atpB*, *atpE*, atpF *, *atpH*, *atpI*
	Cytochrome b/f complex	*petA*, petB *, *petD*, *petG*, *petL*, *petN*
	Cytochrome c synthesis	*ccsA*
	NADPH dehydrogenase	ndhA *, *ndhB* * (×2), *ndhC*, *ndhD*, *ndhE*, *ndhF ^a^*, *ndhG*, *ndhH, ndhI*, *ndhJ*, *ndhK*
Transcription- and translation-related genes	Transcription	*rpoA*, *rpoB*, rpoC1 *, *rpoC2*
	Ribosomal protein	rpl2 * (×2), *rpl14*, rpl16 *, *rpl20*, *rpl23* (×2), *rpl32*, *rpl33*, *rpl36*, *rps2*, *rps3*, *rps4*, *rps7* (×2), *rps8*, *rps11*, rps12 ** (×2, tran-spliced), *rps14*, *rps15*, rps16 *, *rps18*, *rps19*
RNA genes	Ribosomal RNA	*rrn4.5* (×2), *rrn5* (×2), *rrn16* (×2), *rrn23* (×2)
	Transfer RNA	trnA-UGC * (×2), *trnC-GCA, trnD-GUC*, *trnE-UUC*, *trnF-GAA*, *trnfM-CAU*, *trnG-GCC*, trnG-UCC *^b^, *trnH-GUG*, *trnI-CAU* (×2), trnI-GAU * (×2), trnK-UUU *, *trnL-CAA* (×2), trnL-UAA *, *trnL-UAG, trnM-CAU*, *trnN-GUU* (×2), *trnP-UGG*, *trnQ-UUG*, *trnR-ACG* (×2), *trnR-UCU*, *trnS-GCU*, *trnS-GGA*, *trnS-UGA*, *trnT-GGU*, *trnT-UGU*, *trnV-GAC* (×2), trnV-UAC *, *trnW-CCA*, *trnY-GUA*
Miscellaneous group	Maturase	*matK*
	Inner membrane protein	*cemA*
	ATP-dependent protease	clpP1 **
	Acetyl-CoA carboxylase	*accD*
	Unknown functions	*ycf1* (×2) ^c^, *ycf2* (×2), *ycf68* (×2) ^d^

* Number of asterisks (*) indicates the number of introns present in the respective genes. ^a^ The *ndhF* gene of *D. hancei* is truncated. ^b^ Present only in *D. hancei, D. millettii, D. cantenatensis*, and *D. assamica.* Absent in *D. benthamii.* ^c^ One copy is pseudogene. ^d^ Present only in *D. hancei* and *D. millettii*.

**Table 3:** Summary of mitochondrial plastid DNAs identified in the five *Dalbergia* cp genomes.

Species	Number of MTPTs (Range of Length of MTPTs)					Total Length & Percentage of MTPTs
	Total	Protein-Coding	Transfer RNA	Ribosomal RNA	Non-Coding	
*Dalbergia hancei*	45 (50–2493 bp)	12 (50–2493 bp)	9 (54–276 bp)	22 (51-1022 bp)	2 (76–202 bp)	16,376 bp (10.51%)
*Dalbergia millettii*	44 (50–2493 bp)	12 (50–2493 bp)	8 (54–86 bp)	22 (51–1022 bp)	2 (76–278 bp)	16,176 bp (10.38%)
*Dalbergia benthamii*	44 (50–2493 bp)	11 (86–2493 bp)	6 (54–81 bp)	22 (51–1020 bp)	5 (50–279 bp)	15,636 bp (9.98%)
*Dalbergia candenatensis*	44 (50–2493 bp)	11 (86–2493 bp)	6 (54–86 bp)	22 (51–1021 bp)	5 (50–281 bp)	15,640 bp (10.03%)
*Dalbergia assamica*	44 (50–2493 bp)	12 (50–2493 bp)	8 (54–86 bp)	22 (51–1022 bp	2 (76–293 bp)	15,654 bp (10.05%)
*Dalbergia odorifera* (NC_049008.1)	44 (50–2493 bp)	10 (50–2493 bp)	11 (55–193 bp)	21 (51–1022 bp)	2 (109–193 bp)	15,670 bp (10.04%)

**Table 4:** Information of the studied specimens.

Species	Specimen Voucher No.	Collector No.	Inventory No.
*Dalbergia hancei*	D.T.W. Lau 240	D.T.W. Lau 240	CUSLSH2130
*Dalbergia candenatensis*	R & E 016	Rare and endangered plants 016	HK0051279
*Dalbergia assamica*	R & E 020	Rare and endangered plants 020	HK0051283
*Dalbergia millettii*	T.Y. Siu 580	T.Y. Siu 580	CUSLSH2682
*Dalbergia benthamii*	S.K. Tsang 070	S.K. Tsang 070	CUSLSH1909

## Data Availability

The complete chloroplast genome sequences of five *Dalbergia* species were deposited in GenBank at https://www.ncbi.nlm.nih.gov/nuccore (accessed on 23 March 2022), with accession numbers OM328089.1 to OM328093.1.

## Supplementary Materials

The following supporting information can be downloaded at https://www.mdpi.com/article/10.3390/plants11091109/s1: Table S1: Number of reads obtained in NGS and coverage of the five Dalbergia cp genomes; Table S2: Density of simple sequence repeats (SSRs) of the five *Dalbergia* cp genomes; Figure S1: mVISTA visualization of alignment of the five *Dalbergia* cp genomes; Table S3: Detailed information of MTPTs identified by BLASTn; Figure S2: Original (un-condensed) maximum likelihood tree of 46 *Dalbergia* chloroplast genomes; Figure S3: Neighbor-joining trees of the four divergence hotspots; Figure S4: Neighbor-joining tree of *ITS* sequences of *Dalbergia* (condensed at 50%) with *Pterocarpus*, *Glycine*, *Lotus* as outgroup; Figure S5: Alignment of *rps16* amino acid sequences of *Dalbergia* cp genomes; Figure S6: Amino acid sequences of *ycf68* from *Dalbergia* cp genomes; and Table S4: Pairwise K2P distances of *Dalbergia* cp genomes.
