# Supplementary material for: Comparative Analysis of Chloroplast Genomes of Dalbergia Species for Identification and Phylogenetic Analysis
Source: Plants (Basel). 2022 Apr 20;11(9):1109. doi: 10.3390/plants11091109 (PMC9104903; doi:10.3390/plants11091109)
Supplement: Supplementary file 1 [file plants-11-01109-s001.zip › Supp Figure S1 - mVista.pdf]

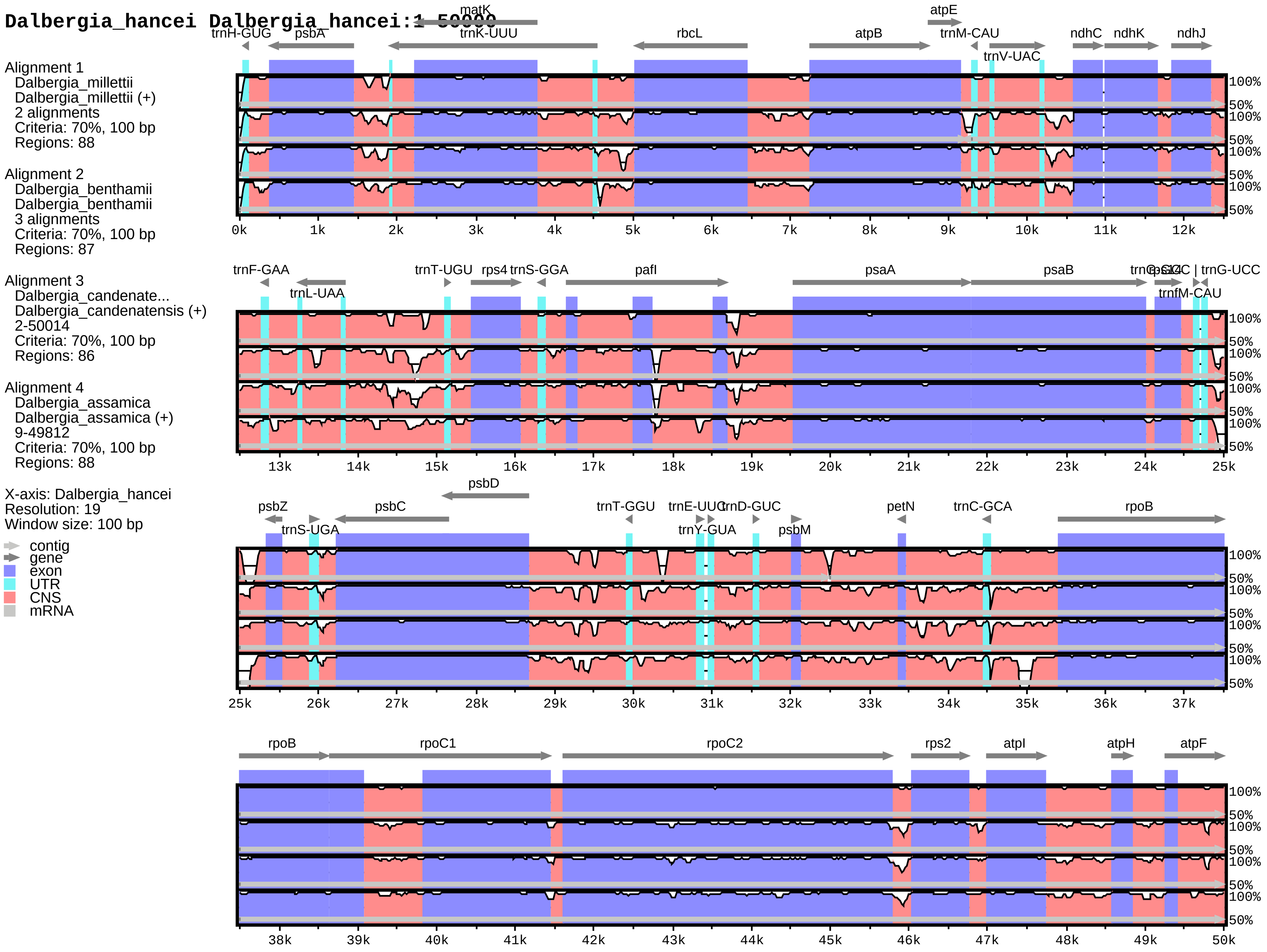
Dalbergia_hancei Dalbergia_hancei:1-50000
Alignment 1
Dalbergia_millettii
Dalbergia_millettii (+)
2 alignments
Criteria: 70%, 100 bp
Regions: 88
Alignment 2
Dalbergia_benthamii
Dalbergia_benthamii
3 alignments
Criteria: 70%, 100 bp
Regions: 87
Alignment 3
Dalbergia_candenate...
Dalbergia_candenatensis (+)
2-50014
Criteria: 70%, 100 bp
Regions: 86
Alignment 4
Dalbergia_assamica
Dalbergia_assamica (+)
9-49812
Criteria: 70%, 100 bp
Regions: 88
X-axis: Dalbergia_hancei
Resolution: 19
Window size: 100 bp
contig
gene
exon
UTR
CNS
mRNA
trnH-GUG
psbA
matK
trnK-UUU
rbcL
atpB
atpE
trnM-CAU
trnV-UAC
ndhC
ndhK
ndhJ
trnF-GAA
trnL-UAA
trnT-UGU
rps4
trnS-GGA
pafI
psaA
psaB
rps14
trnfM-CAU
trnG-UCC
psbZ
trnS-UGA
psbC
psbD
trnT-GGU
trnE-UUC
trnY-GUA
trnD-GUC
psbM
petN
trnC-GCA
rpoB
rpoC1
rpoC2
rps2
atpI
atpH
atpF
100%
50%
0k
1k
2k
3k
4k
5k
6k
7k
8k
9k
10k
11k
12k
13k
14k
15k
16k
17k
18k
19k
20k
21k
22k
23k
24k
25k
26k
27k
28k
29k
30k
31k
32k
33k
34k
35k
36k
37k
38k
39k
40k
41k
42k
43k
44k
45k
46k
47k
48k
49k
50k

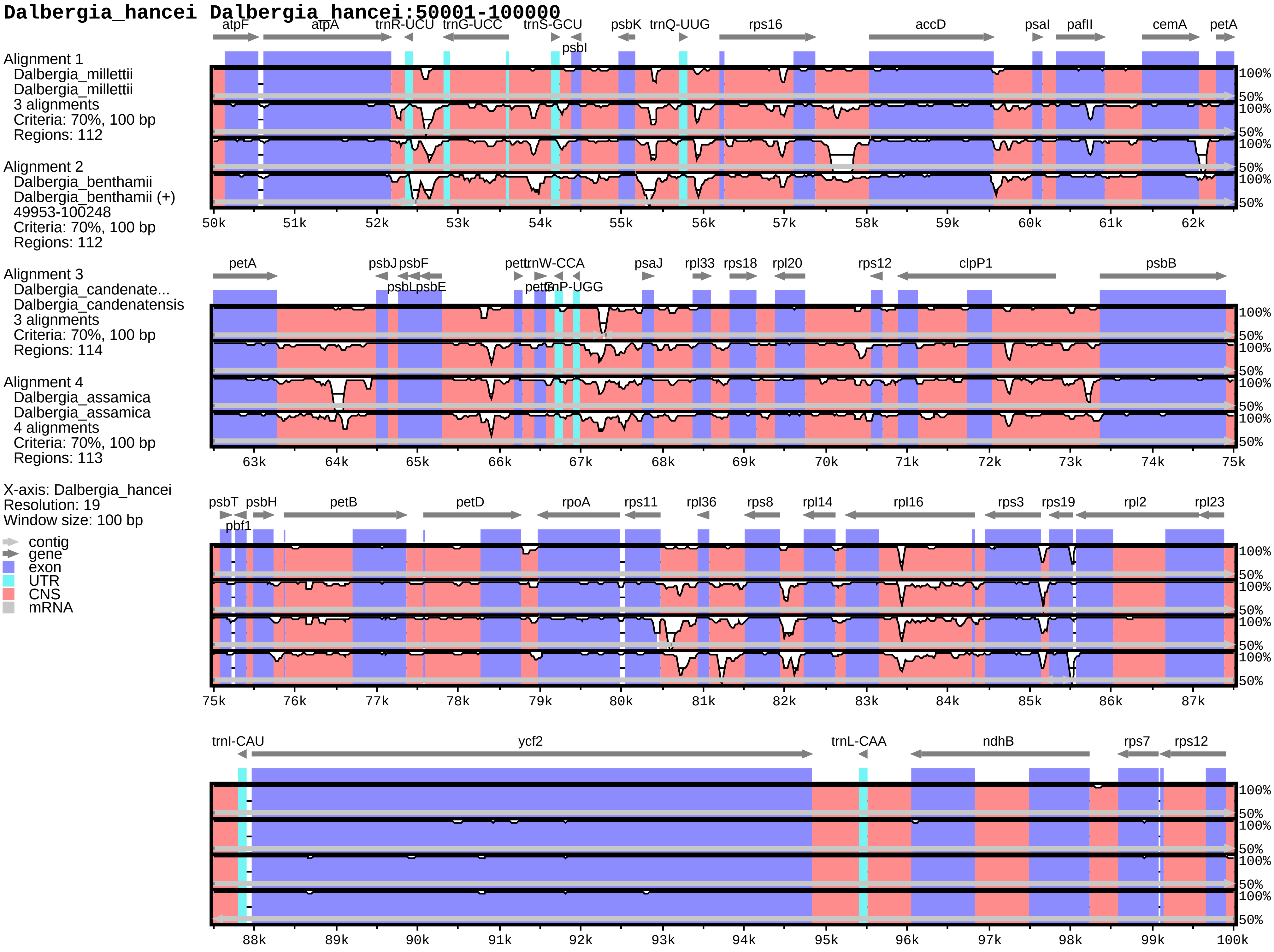
Dalbergia_hancei Dalbergia_hancei:50001-100000
Alignment 1
Dalbergia_millettii
Dalbergia_millettii
3 alignments
Criteria: 70%, 100 bp
Regions: 112
Alignment 2
Dalbergia_benthamii
Dalbergia_benthamii (+)
49953-100248
Criteria: 70%, 100 bp
Regions: 112
Alignment 3
Dalbergia_candenate...
Dalbergia_candenatensis
3 alignments
Criteria: 70%, 100 bp
Regions: 114
Alignment 4
Dalbergia_assamica
Dalbergia_assamica
4 alignments
Criteria: 70%, 100 bp
Regions: 113
X-axis: Dalbergia_hancei
Resolution: 19
Window size: 100 bp
contig
gene
exon
UTR
CNS
mRNA
atpF atpA trnR-UCU trnG-UCC trnS-GCU psbI psbK trnQ-UUG rps16 accD psaI pafII cemA petA
petA psbJ psbF psbL psbE petL trnW-CCA petG trnP-UGG psaJ rpl33 rps18 rpl20 rps12 clpP1 psbB
psbT pbf1 psbH petB petD rpoA rps11 rpl36 rps8 rpl14 rpl16 rps3 rps19 rpl2 rpl23
trnI-CAU ycf2 trnL-CAA ndhB rps7 rps12
100%
50%
50k 51k 52k 53k 54k 55k 56k 57k 58k 59k 60k 61k 62k
63k 64k 65k 66k 67k 68k 69k 70k 71k 72k 73k 74k 75k
75k 76k 77k 78k 79k 80k 81k 82k 83k 84k 85k 86k 87k
88k 89k 90k 91k 92k 93k 94k 95k 96k 97k 98k 99k 100k

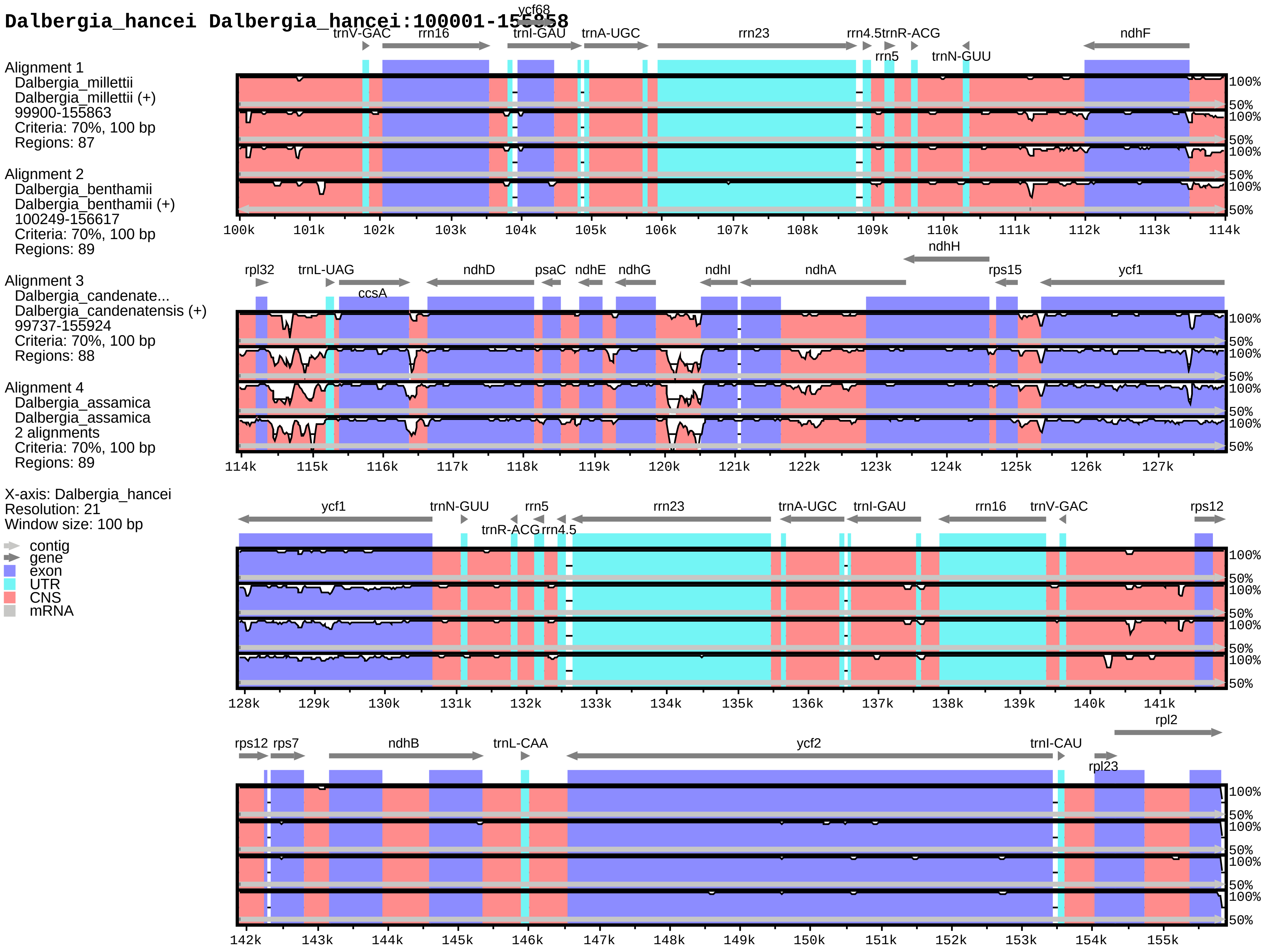

Dalbergia_hancei Dalbergia_hancei:100001-155858
Alignment 1
Dalbergia_millettii
Dalbergia_millettii (+)
99900-155863
Criteria: 70%, 100 bp
Regions: 87
Alignment 2
Dalbergia_benthamii
Dalbergia_benthamii (+)
100249-156617
Criteria: 70%, 100 bp
Regions: 89
Alignment 3
Dalbergia_candenate...
Dalbergia_candenatensis (+)
99737-155924
Criteria: 70%, 100 bp
Regions: 88
Alignment 4
Dalbergia_assamica
Dalbergia_assamica
2 alignments
Criteria: 70%, 100 bp
Regions: 89
X-axis: Dalbergia_hancei
Resolution: 21
Window size: 100 bp
contig
gene
exon
UTR
CNS
mRNA
ycf68
trnV-GAC
rrn16
trnI-GAU
trnA-UGC
rrn23
rrn4.5
trnR-ACG
rrn5
trnN-GUU
ndhF
100%
50%
100k
101k
102k
103k
104k
105k
106k
107k
108k
109k
110k
111k
112k
113k
114k
ndhH
rpl32
trnL-UAG
ccsA
ndhD
psaC
ndhE
ndhG
ndhI
ndhA
rps15
ycf1
114k
115k
116k
117k
118k
119k
120k
121k
122k
123k
124k
125k
126k
127k
ycf1
trnN-GUU
rrn5
trnR-ACG
rrn4.5
rrn23
trnA-UGC
trnI-GAU
rrn16
trnV-GAC
rps12
128k
129k
130k
131k
132k
133k
134k
135k
136k
137k
138k
139k
140k
141k
rpl2
rps12
rps7
ndhB
trnL-CAA
ycf2
trnI-CAU
rpl23
142k
143k
144k
145k
146k
147k
148k
149k
150k
151k
152k
153k
154k
155k
