# Supplementary material for: Comparative Analysis of Chloroplast Genomes of Dalbergia Species for Identification and Phylogenetic Analysis
Source: Plants (Basel). 2022 Apr 20;11(9):1109. doi: 10.3390/plants11091109 (PMC9104903; doi:10.3390/plants11091109)
Supplement: Supplementary file 1 [file plants-11-01109-s001.zip › Supp Figure S5 - Amino acid sequences of rps16 of Dalbergia cp genomes.pdf]

```
                                     10        20        30        40        50        60        70        80        90
                                ....|....|....|....|....|....|....|....|....|....|....|....|....|....|....|....|....|....|.
OM328090.1_Dalbergia_hancei     MVKLRLKRCGKKQRAVYRIVAIDVRSRRGGRDLRKVGFYDPIKNQTHLNIPVILYFLEQGAQPTGTVQDISKRAGVSLELSPTQQTKVN*-
OM328092.1_Dalbergia_millettii  MVKLRLKRCGKKQRAVYRIVAIDVRSRRGGRDLRKVGFYDPIKNQTYFNIPVILYFLEQGAQPTGTVQDISKRAGVSLELSPTQQTKVN*-
OM328091.1_Dalbergia_benthamii  MVKLRLKRCGKKQRAVYRIVAIDVRSRRGGRDLRKVGFYDPIKNQTYLNIPVILYFLEQGAQPIGTVQDISKRAGVSLELSPTQQTKVN*-
OM328093.1_Dalbergia_candenate  MVKLRLKRCGKKQRAVYRIVPIDVRSRRGGRDLRKVGFYDPIKNQTYLNIPVILYFLEQGAQPTGTVQDISKRTGVSLELSPTQQTKVN*-
OM328089.1_Dalbergia_assamica   MVKLRLKRCGKKQRAVYRIVAIDVRSRRAGRDLRKVGFYDPIKNQTYLNIPVILYFLEQGAQPTGTVQNISKRAGVSLELSPTQQTKVN*-
MN251245.1_Dalbergia_hupeana    MVKLRLKRCGKKQRAVYRIVAIDVRSRRGGRYLRKVGFYDPIKNQTYLNIPVILYFLEQGAQPTGTVQDISKRAGVFLELSPTQQTKVN*-
MN714220.1_Dalbergia_hupeana    MVKLRLKRCGKKQRAVYRIVAIDVRSRRAGRDLRKVGFYDPIKNQTYLNIPVILYFLEQGAQPTGTVQNISKRAGVSLELSPTQQTKVN*-
NC_058536.1_Dalbergia_hupeana   MVKLRLKRCGKKQRAVYRIVAIDVRSRRAGRDLRKVGFYDPIKNQTYLNIPVILYFLEQGAQPTGTVQNISKRAGVSLELSPTQQTKVN*-
MN251247.1_Dalbergia_cochinchi  MVKLRLKRCGKKQRAVYRIVAIDVRSRRGGRYLRKVGFYDPIKNQTYLNIPVILYFLEQGAQPTGTVQDISKRAGVFLELSPTQQTKVN*-
NC_058539.1_Dalbergia_cochinch  MVKLRLKRCGKKQRAVYRIVAIDVRSRRGGRYLRKVGFYDPIKNQTYLNIPVILYFLEQGAQPTGTVQDILKRAGVFLELSPTQQTKVN*-
MN251242.1_Dalbergia_sissoo     MVKLRLKRCGKKQRAVYRIVAIDVRSRRGGRYLRKVGFYDPIKNQTYLNIPVILYFLEQGAQPTGTVQDISKRAGVSLELSPTQQTKVN*-
NC_049043.1_Dalbergia_sissoo    MVKLRLKRCGKKQRAVYRIVAIDVRSRRGGRDLRKVGFYDPIKNQTYLNIPLILYFLEQGAQPTGTVQDISKRAGVSLELSPTQQTKVN*-
MN714222.1_Dalbergia_sissoo     MVKLRLKRCGKKQRAVYRIVAIDVRSRRGGRDLRKVGFYDPIKNQTYLNIPLILYFLEQGAQPTGTVQDISKRAGVSLELSPTQQTKVN*-
NC_049044.1_Dalbergia_frutesce  MVKLRLKRCGKKQRAVYRIVAIDVRSRRGGRDLRKVGFYDPIKNQTYLNIPVILYFLEQGAQPTGTVQDISKRAGVSLELSPTQQTKVN*-
NC_058537.1_Dalbergia_nigra     MVKLRLKRCGKKQR-----------------------------------------------------------------------------
MF668133.1_Dalbergia_odorifera  MVKLRLKRCGKKQRAVYRIVAIDVRSRRGGRDLRKVGFYDPIKNQTYLNIPLILYFLEQGAQPTGTVQDILKRAGVSLELSPTQQTKVN*-
MN251244.1_Dalbergia_odorifera  MVKLRLKRCGKKQRAVYRIVAIDVRSRRGGRDLRKVGFYDPIKNQTYLNIPLILYFLEQGAQPTGTVQDILKRAGVSLELSPTQQTKVN*-
MN202181.1_Dalbergia_odorifera  MVKLRLKRCGKKQRAVYRIVAIDVRSRRGGRDLRKVGFYDPIKNQTYLNIPLILYFLEQGAQPTGTVQDILKRAGVSLELSPTQQTKVN*-
NC_049008.1_Dalbergia_odorifer  MVKLRLKRCGKKQRAVYRIVAIDVRSRRGGRDLRKVGFYDPIKNQTYLNIPLILYFLEQGAQPTGTVQDILKRAGVSLELSPTQQTKVN*-
MT644131.1_Dalbergia_odorifera  MVKLRLKRCGKKQRAVYRIVAIDVRSRRGGRDLRKVGFYDPIKNQTYLNIPLILYFLEQGAQPTGTVQDILKRAGVSLELSPTQQTKVN*-
MN833948.1_Dalbergia_hancei     MVKLRLKRCGKKQRAVYRIVAIDVRSRRGGRDLRKVGFYDTIKNQTYFNIPVILYFLEQGAQPTGTVQDISKRAGVSLELSPTQQTKVN*-
MN251241.1_Dalbergia_tonkinens  MVKLRLKRCGKKQRAVYRIVAIDVRSRRGGRDLRKVGFYDPIKNQTYLNIPLILYFLEQGAQPTGTVQDILKRAGVSLELSPTQQTKVN*-
MN202182.1_Dalbergia_tonkinens  MVKLRLKRCGKKQRAVYRIVAIDVRSRRGGRDLRKVGFYDPIKNQTYLNIPLILYFLEQGAQPTGTVQDILKRAGVSLELSPTQQTKVN*-
NC_049007.1_Dalbergia_tonkinen  MVKLRLKRCGKKQRAVYRIVAIDVRSRRGGRDLRKVGFYDPIKNQTYLNIPLILYFLEQGAQPTGTVQDILKRAGVSLELSPTQQTKVN*-
MK599470.1_Dalbergia_tonkinens  MVKLRLKRCGKKQRAVYRIVAIDVRSRRGGRDLRKVGFYDPIKNQTYLNIPLILYFLEQGAQPTGTVQDILKRAGVSLELSPTQQTKVN*-
MT644133.1_Dalbergia_tonkinens  MVKLRLKRCGKKQRAVYRIVAIDVRSRRGGRDLRKVGFYDPIKNQTYLNIPLILYFLEQGAQPTGTVQDILKRAGVSLELSPTQQTKVN*-
MN714219.1_Dalbergia_obtusifol  MVKLRLKRCGKKQRAVYRIVAIDVRSRRGGRYLRKVGFYDPIKNQTYLNIPVILYFLERGAQPTGTVQDISKRADVSLELSPTQQTKVN*-
NC_049048.1_Dalbergia_armata    MVKLRLKRCGKKQRAVYRIVAIDVRSRRGGRDLRKVGFYDPIKNQTYLNIPVILYFLEQGAQPTGTVQDISKRAGVSLELSPTQQTKVN*-
MN251249.1_Dalbergia_balansae   MVKLRLKRCGKKQRAVYRIVAIDVRSRRAGRDLRKVGFYDPIKNQTYLNIPVILYFLEQGAQPTGTVQNISKRAGVSLELSPTQQTKVN*-
MN905599.1_Dalbergia_balansae   MVKLRLKRCGKKQRAVYRIVAIDVRSRRAGRDLRKVGFYDPIKNQTYLNIPVILYFLEQGAQPTGTVQNISKRAGVSLELSPTQQTKVN*-
MN714221.1_Dalbergia_mimosoide  MVKLRLKRCGKKQRAVYRIVAIDVRSRRGGRDLRKVGFYDPIKNQTYLNIPVILYFLEQGAQPTGTVQDISKRAGVSLELSPTQQTKVN*-
MK599253.2_Dalbergia_cultrata   MVKLRLKRCGKKQRAVYRIVAIDVRSRRGGRDLRKVGFYDPIKNQTYLNIPVILYFLEQGAQPTGTVQDISKRAGVSLELSPTQQTKVN*-
MT644128.1_Dalbergia_cultrata   MVKLRLKRCGKKQRAVYRIVAIDVRSRRGGRDLRKVGFYDPIKNQTYLNIPVILYFLEQGAQPTGTVQDISKRAGVSLELSPTQQTKVN*-
MN251243.1_Dalbergia_oliveri    MVKLRLKRCGKKQRAIYRIVAIDVRSRRGGRDLRKVGFYDPIKNQTYLNIPVILYFLEQGAQPTGTVQDISKRAGVSLELSPTQQTKKVN*
MN823694.1_Dalbergia_oliveri    MVKLRLKRCGKKQRAVYRIVAIDVRSRRGGRDLRKVGFYDPIKNQTYLNIPVILYFLEQGAQPTGTVQDISKRAGVSLELSPTQQTKVN*-
MT644132.1_Dalbergia_oliveri    MVKLRLKRCGKKQRAIYRIVAIDVRSRRGGRDLRKVGFYDPIKNQTYLNIPVILYFLEQGAQPTGTVQDISKRAGVSLELSPTQQTKKVN*
NC_049045.1_Dalbergia_obovata   MVKLRLKRCGKKQRAVYRIVAIDVRSRRGGRDLRKVGFYDPIKNQTYLNIPVILYFLEQGAQPTGTVQDISKRAGVSLELSPTQQTKVN*-
NC_049046.1_Dalbergia_cearensi  MVKLRLKRCGKKQRAVYRIVAIDVRSRRGGRDLRKVGFYDPIKNQTYLNIPVILYFLEQGAQPTGTVQDISKRAGVSLELSPTQQTKVN*-
NC_049049.1_Dalbergia_martinii  MVKLRLKRCGKKQRAVYRIVAIDVRSRRGGRDLRKVGFYDPIKNQTYLNIPVILYFLEQGAQPTGTVQDISKRAGVSLELSPTQQTKVN*-
MN251246.1_Dalbergia_hainanens  MVKLRLKRCGKKQRAVYRIVAIDVRSRRGGRDLRKVGFYDPIKNQTYLNIPVILYFLEQNRALNLQELFRIFQKGPVFLWNFPLLNKRKLI
MN202184.1_Dalbergia_yunnanens  MVKLRLKRCGKKQRAVYRIVAIDVRSRRGGRDLRKVGFYDPIKNKTYLNIPLILYFLEQGAQPTGTVQDISKRAGVSLELSPTQQTKVN*-
NC_049009.1_Dalbergia_yunnanen  MVKLRLKRCGKKQRAVYRIVAIDVRSRRGGRDLRKVGFYDPIKNQTYLTSYSIFP*TGRSTYRNCSGYFKKGRCFFGTFPYSTNES*L---
MN251248.1_Dalbergia_bariensis  MVKLRLKRCGKKQRAVYRILAIDVRSRRGGRDLRKVGFYDPIKNQTYLNIPVILYFLEQGAQPTGTVQDISKRAGVSLELSPTQQTKVN*-
NC_058538.1_Dalbergia_bariensi  MVKLRLKRCGKKQRAVYRILAIDVRSRRGGRDLRKVGFYDPIKNQTYLNIPVILYFLEQGAQPTGTVQDISKRAGVSLELSPTQQTKVN*-
NC_049047.1_Dalbergia_chloroca  MVKLRLKRCGKKQRAVYRIVAIDVRSRRGGRDLRKVGFYDPIKNQTYLNIPVILYFLEQGAQPTGTVQDISKRAGVSLELSPTQQTKVN*-
NC_049050.1_Dalbergia_vietname  MVKLRLKRCGKKQRAVYRIVAIDVRSRRGGRDLRKVGFYDPIKNQTYLNIPLILYFLEQGAQPTGTVQDISKRAGVSLELSPTQQTKVN*-
```
