# Supplementary material for: Comparative Analysis of Chloroplast Genomes of Dalbergia Species for Identification and Phylogenetic Analysis
Source: Plants (Basel). 2022 Apr 20;11(9):1109. doi: 10.3390/plants11091109 (PMC9104903; doi:10.3390/plants11091109)
Supplement: Supplementary file 1 [file plants-11-01109-s001.zip › Supp Figure S6 - Amino acid sequences of ycf68 from Dalbergia cp genomes.pdf]

```
                                       10        20        30        40        50        60        70        80        90       100
                                ....|....|....|....|....|....|....|....|....|....|....|....|....|....|....|....|....|....|....|....|
OM328091.1_Dalbergia_benthamii  MAYFSCSNRGLV*NQTQVRSNVDPTFYSLVGSGRSGGDHHGSSLLENSYIPYQYMDSYLSSTGLGSASMEKEKRST*QRIFTDQELRDRPFHSGVTARSY
OM328093.1_Dalbergia_candenate  MAYFSCSNRGLV*NQTQVRSNVDPTFSSLVGSGRSGGDHHGSSLLENSYIPYQYMDSYLSSTGLGSASMEKEKRST*QRIFTDQELRDRPFHSGVTARSY
OM328089.1_Dalbergia_assamica   MAYFSCSNRGLKPNSGEIQCRSNFLFTRGIRAVRGGPPRLLSSREFIHPLSVYGQLSLEHRFRFGLNGKRKTEHLTTYLHRPRTTRSPLSFWGDGEIVPF
MN714220.1_Dalbergia_hupeana    MAYFSCSNRGLKPNSGEIQCRSNFLFTRGIRAVRGGPPRLLSSREFIHPLSVYGQLSLEHRFRFGLNGKRKTEHLTTYLHRPRTTRSPLSFWGDGEIVPF
NC_058536.1_Dalbergia_hupeana   MAYFSCSNRGLKPNSGEIQCRSNFLFTRGIRAVRGGPPRLLSSREFIHPLSVYGQLSLEHRFRFGLNGKRKTEHLTTYLHRPRTTRSPLSFWGDGEIVPF
MN251249.1_Dalbergia_balansae   MAYFSCSNRGLKPNSGEIQCRSNFLFTRGIRAVRGGPPRLLSSREFIHPLSVYGQLSLEHRFRFGLNGKRKTEHLTTYLHRPRTTRSPLSFWGDGEIVPF
MN905599.1_Dalbergia_balansae   MAYFSCSNRGLKPNSGEIQCRSNFLFTRGIRAVRGGPPRLLSSREFIHPLSVYGQLSLEHRFRFGLNGKRKTEHLTTYLHRPRTTRSPLSFWGDGEIVPF
OM328090.1_Dalbergia_hancei     MAYFSCSNRGLKPNSGEIQCRSNFLFTRGIRAVRGGPPRLLSSREFIHPLSVYGQLSLEHRFRFGLNGKRKTEHLTTYLHRPRTTRSPLSFWGDGEIVPF
OM328092.1_Dalbergia_millettii  MAYFSCSNRGLKPNSGEIQCRSNFLFTRGIRAVRGGPPRLLSSREFIHPLSVYGQLSLEHRFRFGLNGKRKTEHLTTYLHRPRTTRSPLSFWGDGEIVPF
MN251245.1_Dalbergia_hupeana    MAYFSCSNRGLKPNSGEIQCRSNFLFTRGIRAVRGGPPRLLSSREFIHPLSVYGQLSLEHRFRFGLNGKRKTEHLTTYLHRPRTTRSPLSFWGDGEIVPF
MN251247.1_Dalbergia_cochinchi  MAYFSCSNRGLKPNSGEIQCRSNFLFTRGIRAVRGGPPRLLSSREFIHPLSVYGQLSLEHRFRFGLNGKRKTEHLTTYLHRPRTTRSPLSFWGDGEIVPF
NC_058539.1_Dalbergia_cochinch  MAYFSCSNRGLKPNSGEIQCRSNFLFTRGIRAVRGGPPRLLSSREFIHPLSVYGQLSLEHRFRFGLNGKRKTEHLTTYLHRPRTTRSPLSFWGDGEIVPF
MN251242.1_Dalbergia_sissoo     MAYFSCSNRGLKPNSGEIQCRSNFLFTRGIRAVRGGPPRLLSSREFIHPLSVYGQLSLEHRFRFGLNGKRKTEHLTTYLHRPRTTRSPLSFWGDGEIVPF
NC_049043.1_Dalbergia_sissoo    MAYFSCSNRGLKPNSGEIQCRSNFLFTRGIRAVRGGPPRLLSSREFIHPLSVYGQLSLEHRFRFGLNGKRKTEHLTTYLHRPRTTRSPLSFWGDGEIVPF
MN714222.1_Dalbergia_sissoo     MAYFSCSNRGLKPNSGEIQCRSNFLFTRGIRAVRGGPPRLLSSREFIHPLSVYGQLSLEHRFRFGLNGKRKTEHLTTYLHRPRTTRSPLSFWGDGEIVPF
NC_049044.1_Dalbergia_frutesce  MAYFSCSNRGLKPNSGEIQCRSNFLFTRGIRAVRGGPPRLLSSREFIHPLSVYGQLSLEHRFRFGLNGKRKTEHLTTYLHRPRTTRSPLSFWGDGEIVPF
NC_058537.1_Dalbergia_nigra     MAYFSCSNRGLKPNSGEIQCRSNFLFTRGIRAVRGGPPRLLSSREFIHPLSVYGQLSLEHRFRFGLNGKRKTEHLTTYLHRPRTTRSPLSFWGDGEIVPF
MF668133.1_Dalbergia_odorifera  MAYFSCSNRGLKPNSGEIQCRSNFLFTRGIRAVRGGPPRLLSSREFIHPLSVYGQLSLEHRFRFGLNGKRKTEHLTTYLHRPRTTRSPLSFWGDGEIVPF
MN251244.1_Dalbergia_odorifera  MAYFSCSNRGLKPNSGEIQCRSNFLFTRGIRAVRGGPPRLLSSREFIHPLSVYGQLSLEHRFRFGLNGKRKTEHLTTYLHRPRTTRSPLSFWGDGEIVPF
MN202181.1_Dalbergia_odorifera  MAYFSCSNRGLKPNSGEIQCRSNFLFTRGIRAVRGGPPRLLSSREFIHPLSVYGQLSLEHRFRFGLNGKRKTEHLTTYLHRPRTTRSPLSFWGDGEIVPF
NC_049008.1_Dalbergia_odorifer  MAYFSCSNRGLKPNSGEIQCRSNFLFTRGIRAVRGGPPRLLSSREFIHPLSVYGQLSLEHRFRFGLNGKRKTEHLTTYLHRPRTTRSPLSFWGDGEIVPF
MT644131.1_Dalbergia_odorifera  MAYFSCSNRGLKPNSGEIQCRSNFLFTRGIRAVRGGPPRLLSSREFIHPLSVYGQLSLEHRFRFGLNGKRKTEHLTTYLHRPRTTRSPLSFWGDGEIVPF
MN833948.1_Dalbergia_hancei     MAYFSCSNRGLKPNSGEIQCRSNFLFTRGIRAVRGGPPRLLSSREFIHPLSVYGQLSLEHRFRFGLNGKRKTEHLTTYLHRPRTTRSPLSFWGDGEIVPF
MN251241.1_Dalbergia_tonkinens  MAYFSCSNRGLKPNSGEIQCRSNFLFTRGIRAVRGGPPRLLSSREFIHPLSVYGQLSLEHRFRFGLNGKRKTEHLTTYLHRPRTTRSPLSFWGDGEIVPF
MN202182.1_Dalbergia_tonkinens  MAYFSCSNRGLKPNSGEIQCRSNFLFTRGIRAVRGGPPRLLSSREFIHPLSVYGQLSLEHRFRFGLNGKRKTEHLTTYLHRPRTTRSPLSFWGDGEIVPF
NC_049007.1_Dalbergia_tonkinen  MAYFSCSNRGLKPNSGEIQCRSNFLFTRGIRAVRGGPPRLLSSREFIHPLSVYGQLSLEHRFRFGLNGKRKTEHLTTYLHRPRTTRSPLSFWGDGEIVPF
MK599470.1_Dalbergia_tonkinens  MAYFSCSNRGLKPNSGEIQCRSNFLFTRGIRAVRGGPPRLLSSREFIHPLSVYGQLSLEHRFRFGLNGKRKTEHLTTYLHRPRTTRSPLSFWGDGEIVPF
MT644133.1_Dalbergia_tonkinens  MAYFSCSNRGLKPNSGEIQCRSNFLFTRGIRAVRGGPPRLLSSREFIHPLSVYGQLSLEHRFRFGLNGKRKTEHLTTYLHRPRTTRSPLSFWGDGEIVPF
MN714219.1_Dalbergia_obtusifol  MAYFSCSNRGLKPNSGEIQCRSNFLFTRGIRAVRGGPPRLLSSREFIHPLSVYGQLSLEHRFRFGLNGKRKTEHLTTYLHRPRTTRSPLSFWGDGEIVPF
NC_049048.1_Dalbergia_armata    MAYFSCSNRGLKPNSGEIQCRSNFLFTRGIRAVRGGPPRLLSSREFIHPLSVYGQLSLEHRFRFGLNGKRKTEHLTTYLHRPRTTRSPLSFWGDGEIVPF
MN714221.1_Dalbergia_mimosoide  MAYFSCSNRGLKPNSGEIQCRSNFLFTRGIRAVRGGPPRLLSSREFIHPLSVYGQLSLEHRFRFGLNGKRKTEHLTTYLHRPRTTRSPLSFWGDGEIVPF
MK599253.2_Dalbergia_cultrata   MAYFSCSNRGLKPNSGEIQCRSNFLFTRGIRAVRGGPPRLLSSREFIHPLSVYGQLSLEHRFRFGLNGKRKTEHLTTYLHRPRTTRSPLSFWGDGEIVPF
MT644128.1_Dalbergia_cultrata   MAYFSCSNRGLKPNSGEIQCRSNFLFTRGIRAVRGGPPRLLSSREFIHPLSVYGQLSLEHRFRFGLNGKRKTEHLTTYLHRPRTTRSPLSFWGDGEIVPF
MN251243.1_Dalbergia_oliveri    MAYFSCSNRGLKPNSGEIQCRSNFLFTRGIRAVRGGPPRLLSSREFIHPLSVYGQLSLEHRFRFGLNGKRKTEHLTTYLHRPRTTRSPLSFWGDGEIVPF
MN823694.1_Dalbergia_oliveri    MAYFSCSNRGLKPNSGEIQCRSNFLFTRGIRAVRGGPPRLLSSREFIHPLSVYGQLSLEHRFRFGLNGKRKTEHLTTYLHRPRTTRSPLSFWGDGEIVPF
MT644132.1_Dalbergia_oliveri    MAYFSCSNRGLKPNSGEIQCRSNFLFTRGIRAVRGGPPRLLSSREFIHPLSVYGQLSLEHRFRFGLNGKRKTEHLTTYLHRPRTTRSPLSFWGDGEIVPF
NC_049045.1_Dalbergia_obovata   MAYFSCSNRGLKPNSGEIQCRSNFLFTRGIRAVRGGPPRLLSSREFIHPLSVYGQLSLEHRFRFGLNGKRKTEHLTTYLHRPRTTRSPLSFWGDGEIVPF
NC_049046.1_Dalbergia_cearensi  MAYFSCSNRGLKPNSGEIQCRSNFLFTRGIRAVRGGPPRLLSSREFIHPLSVYGQLSLEHRFRFGLNGKRKTEHLTTYLHRPRTTRSPLSFWGDGEIVPF
NC_049049.1_Dalbergia_martinii  MAYFSCSNRGLKPNSGEIQCRSNFLFTRGIRAVRGGPPRLLSSREFIHPLSVYGQLSLEHRFRFGLNGKRKTEHLTTYLHRPRTTRSPLSFWGDGEIVPF
MN251246.1_Dalbergia_hainanens  MAYFSCSNRGLKPNSGEIQCRSNFLFTRGIRAVRGGPPRLLSSREFIHPLSVYGQLSLEHRFRFGLNGKRKTEHLTTYLHRPRTTRSPLSFWGDGEIVPF
MN202184.1_Dalbergia_yunnanens  MAYFSCSNRGLKPNSGEIQCRSNFLFTRGIRAVRGGPPRLLSSREFIHPLSVYGQLSLEHRFRFGLNGKRKTEHLTTYLHRPRTTRSPLSFWGDGEIVPF
NC_049009.1_Dalbergia_yunnanen  MAYFSCSNRGLKPNSGEIQCRSNFLFTRGIRAVRGGPPRLLSSREFIHPLSVYGQLSLEHRFRFGLNGKRKTEHLTTYLHRPRTTRSPLSFWGDGEIVPF
MN251248.1_Dalbergia_bariensis  MAYFSCSNRGLKPNSGEIQCRSNFLFTRGIRAVRGGPPRLLSSREFIHPLSVYGQLSLEHRFRFGLNGKRKTEHLTTYLHRPRTTRSPLSFWGDGEIVPF
NC_058538.1_Dalbergia_bariensi  MAYFSCSNRGLKPNSGEIQCRSNFLFTRGIRAVRGGPPRLLSSREFIHPLSVYGQLSLEHRFRFGLNGKRKTEHLTTYLHRPRTTRSPLSFWGDGEIVPF
NC_049047.1_Dalbergia_chloroca  MAYFSCSNRGLKPNSGEIQCRSNFLFTRGIRAVRGGPPRLLSSREFIHPLSVYGQLSLEHRFRFGLNGKRKTEHLTTYLHRPRTTRSPLSFWGDGEIVPF
NC_049050.1_Dalbergia_vietname  MAYFSCSNRGLKPNSGEIQCRSNFLFTRGIRAVRGGPPRLLSSREFIHPLSVYGQLSLEHRFRFGLNGKRKTEHLTTYLHRPRTTRSPLSFWGDGEIVPF

                                      110       120       130       140       150       160       170       180
                                ....|....|....|....|....|....|....|....|....|....|....|....|....|....|....|....|...
OM328091.1_Dalbergia_benthamii  HSSLFFMLFPGVWRKLQSIGFS*SSLPERKNVKFFFLSAGTRRLDLAVRRIECLAX~~~~~~~~~~~~~~~~~~~~~~~~~~~
OM328093.1_Dalbergia_candenate  HSSLFFMLFPGVWRKLQSIGFS*SSLPERKNVKFFFLSAGTRRLDLAVRRIECLAX~~~~~~~~~~~~~~~~~~~~~~~~~~~
OM328089.1_Dalbergia_assamica   EPFFHAFPGGLERIAINRIFLILPSRKEEREILFPFRRDQEIGSSRKKNAWLINNSLLGLRPPQSLRTPPIGAMGCVYLSIS*
MN714220.1_Dalbergia_hupeana    EPFFHAFPGGLERIAINRIFLILPSRKEEREILFPFRRDQEIGSSRKKNAWLINNSLLGLRPPQSLRTPPIGAMGCVYLSIS*
NC_058536.1_Dalbergia_hupeana   EPFFHAFPGGLERIAINRIFLILPSRKEEREILFPFRRDQEIGSSRKKNAWLINNSLLGLRPPQSLRTPPIGAMGCVYLSIS*
MN251249.1_Dalbergia_balansae   EPFFHAFPGGLERIAINRIFLILPSRKEEREILFPFRRDQEIGSSRKKNAWLINNSLLGLRPPQSLRTPPIGAMGCVYLSIS*
MN905599.1_Dalbergia_balansae   EPFFHAFPGGLERIAINRIFLILPSRKEEREILFPFRRDQEIGSSRKKNAWLINNSLLGLRPPQSLRTPPIGAMGCVYLSIS*
OM328090.1_Dalbergia_hancei     EPFFHAFPGGLEKIAINRIFLILPSRKEEREILFPFRRDQEIGSSRKKNRMLG*~~~~~~~~~~~~~~~~~~~~~~~~~~~~~~
OM328092.1_Dalbergia_millettii  EPFFHAFPGGLEKIAINRIFLILPSRKEEREILFPFRRDQEIGSSRKKNRMLG*~~~~~~~~~~~~~~~~~~~~~~~~~~~~~~
MN251245.1_Dalbergia_hupeana    EPFFHAFPGGLEKIAINRIFLILPSRKEEREILFPFRRDQEIGSSRKKNRMLG*~~~~~~~~~~~~~~~~~~~~~~~~~~~~~~
MN251247.1_Dalbergia_cochinchi  EPFFHAFPGGLEKIAINRIFLILPSRKEEREILFPFRRDQEIGSSRKKNRMLG*~~~~~~~~~~~~~~~~~~~~~~~~~~~~~~
NC_058539.1_Dalbergia_cochinch  EPFFHAFPGGLEKIAINRIFLILPSRKEEREILFPFRRDQEIGSSRKKNRMLG*~~~~~~~~~~~~~~~~~~~~~~~~~~~~~~
MN251242.1_Dalbergia_sissoo     EPFFHAFPGGLEKIAINRIFLILPSRKEEREILFPFRRDQEIGSSRKKNRMLG*~~~~~~~~~~~~~~~~~~~~~~~~~~~~~~
NC_049043.1_Dalbergia_sissoo    EPFFHAFPGGLEKIAINRIFLILPSRKEEREILFPFRRDQEIGSSRKKNRMLG*~~~~~~~~~~~~~~~~~~~~~~~~~~~~~~
MN714222.1_Dalbergia_sissoo     EPFFHAFPGGLEKIAINRIFLILPSRKEEREILFPFRRDQEIGSSRKKNRMLG*~~~~~~~~~~~~~~~~~~~~~~~~~~~~~~
NC_049044.1_Dalbergia_frutesce  EPFFHAFPGGLEKIAINRIFLILPSRKEEREIVFPFRRDQEIGSSRKKNRMLG*~~~~~~~~~~~~~~~~~~~~~~~~~~~~~~
NC_058537.1_Dalbergia_nigra     EPFFHAFPGGLEKIAINRIFLILPSRKEEREILFPFRRDQEIGSSRKKNRMLG*~~~~~~~~~~~~~~~~~~~~~~~~~~~~~~
MF668133.1_Dalbergia_odorifera  EPFFHAFPGGLEKIAINRIFLILPSRKEEREILFPFRRDQEIGSSRKKNRMLG*~~~~~~~~~~~~~~~~~~~~~~~~~~~~~~
MN251244.1_Dalbergia_odorifera  EPFFHAFPGGLEKIAINRIFLILPSRKEEREILFPFRRDQEIGSSRKKNRMLG*~~~~~~~~~~~~~~~~~~~~~~~~~~~~~~
MN202181.1_Dalbergia_odorifera  EPFFHAFPGGLEKIAINRIFLILPSRKEEREILFPFRRDQEIGSSRKKNRMLG*~~~~~~~~~~~~~~~~~~~~~~~~~~~~~~
NC_049008.1_Dalbergia_odorifer  EPFFHAFPGGLEKIAINRIFLILPSRKEEREILFPFRRDQEIGSSRKKNRMLG*~~~~~~~~~~~~~~~~~~~~~~~~~~~~~~
MT644131.1_Dalbergia_odorifera  EPFFHAFPGGLEKIAINRIFLILPSRKEEREILFPFRRDQEIGSSRKKNRMLG*~~~~~~~~~~~~~~~~~~~~~~~~~~~~~~
MN833948.1_Dalbergia_hancei     EPFFHAFPGGLEKIAINRIFLILPSRKEEREILFPFRRDQEIGSSRKKNRMLG*~~~~~~~~~~~~~~~~~~~~~~~~~~~~~~
MN251241.1_Dalbergia_tonkinens  EPFFHAFPGGLEKIAINRIFLILPSRKEEREILFPFRRDQEIGSSRKKNRMLG*~~~~~~~~~~~~~~~~~~~~~~~~~~~~~~
MN202182.1_Dalbergia_tonkinens  EPFFHAFPGGLEKIAINRIFLILPSRKEEREILFPFRRDQEIGSSRKKNRMLG*~~~~~~~~~~~~~~~~~~~~~~~~~~~~~~
NC_049007.1_Dalbergia_tonkinen  EPFFHAFPGGLEKIAINRIFLILPSRKEEREILFPFRRDQEIGSSRKKNRMLG*~~~~~~~~~~~~~~~~~~~~~~~~~~~~~~
MK599470.1_Dalbergia_tonkinens  EPFFHAFPGGLEKIAINRIFLILPSRKEEREILFPFRRDQEIGSSRKKNRMLG*~~~~~~~~~~~~~~~~~~~~~~~~~~~~~~
MT644133.1_Dalbergia_tonkinens  EPFFHAFPGGLEKIAINRIFLILPSRKEEREILFPFRRDQEIGSSRKKNRMLG*~~~~~~~~~~~~~~~~~~~~~~~~~~~~~~
MN714219.1_Dalbergia_obtusifol  EPFFHAFPGGLEKIAINRIFLILPSRKEEREILFPFHRDQEIGSSRKKNRMLG*~~~~~~~~~~~~~~~~~~~~~~~~~~~~~~
NC_049048.1_Dalbergia_armata    EPFFHAFPGGLEKIAINRIFLILPSRKEEREILFPFRRDQEIGSSRKKNRMLG*~~~~~~~~~~~~~~~~~~~~~~~~~~~~~~
MN714221.1_Dalbergia_mimosoide  EPFFHAFPGGLEKIAINRIFLILPSRKEEREILFPFRRDQEIGSSRKKNRMLG*~~~~~~~~~~~~~~~~~~~~~~~~~~~~~~
MK599253.2_Dalbergia_cultrata   EPFFHAFPGGLEKIAINRIFLILPSRKEEREILFPFRRDQEIGSSRKKNRMLG*~~~~~~~~~~~~~~~~~~~~~~~~~~~~~~
MT644128.1_Dalbergia_cultrata   EPFFHAFPGGLEKIAINRIFLILPSRKEEREILFPFRRDQEIGSSRKKNRMLG*~~~~~~~~~~~~~~~~~~~~~~~~~~~~~~
MN251243.1_Dalbergia_oliveri    EPFFHAFPGGLEKIAINRIFLILPSRKEEREILFPFRRDQEIGSSRKKNRMLG*~~~~~~~~~~~~~~~~~~~~~~~~~~~~~~
MN823694.1_Dalbergia_oliveri    EPFFHAFPGGLEKIAINRIFLILPSRKEEREILFPFRRDQEIGSSRKKNRMLG*~~~~~~~~~~~~~~~~~~~~~~~~~~~~~~
MT644132.1_Dalbergia_oliveri    EPFFHAFPGGLEKIAINRIFLILPSRKEEREILFPFRRDQEIGSSRKKNRMLG*~~~~~~~~~~~~~~~~~~~~~~~~~~~~~~
NC_049045.1_Dalbergia_obovata   EPFFHAFPGGLEKIAINRIFLILPSRKEEREILFPFRRDQEIGSSRKKNRMLG*~~~~~~~~~~~~~~~~~~~~~~~~~~~~~~
NC_049046.1_Dalbergia_cearensi  EPFFHAFPGGLEKIAINRIFLILPSRKEEREIVFPFRRDQEIGSSRKKNRMLG*~~~~~~~~~~~~~~~~~~~~~~~~~~~~~~
NC_049049.1_Dalbergia_martinii  EPFFHAFPGGLEKIAINRIFLILPSRKEEREILFPFRRDQEIGSSRKKNRMLG*~~~~~~~~~~~~~~~~~~~~~~~~~~~~~~
MN251246.1_Dalbergia_hainanens  EPFFHAFPGGLERIAINRIFLILPSRKEEREILFPFRRDQEIGSSRKKNRMLG*~~~~~~~~~~~~~~~~~~~~~~~~~~~~~~
MN202184.1_Dalbergia_yunnanens  EPFFHAFPGGLEKIAINRIFLILPSRKEEREILFPFRRDQEIGSSRKKNRMLG*~~~~~~~~~~~~~~~~~~~~~~~~~~~~~~
NC_049009.1_Dalbergia_yunnanen  EPFFHAFPGGLEKIAINRIFLILPSRKEEREILFPFRRDQEIGSSRKKNRMLG*~~~~~~~~~~~~~~~~~~~~~~~~~~~~~~
MN251248.1_Dalbergia_bariensis  EPFFHAFPGGLEKIAINRIFLILPSRKEEREILFPFRRDQEIGSSRKKNRMLG*~~~~~~~~~~~~~~~~~~~~~~~~~~~~~~
NC_058538.1_Dalbergia_bariensi  EPFFHAFPGGLEKIAINRIFLILPSRKEEREILFPFRRDQEIGSSRKKNRMLG*~~~~~~~~~~~~~~~~~~~~~~~~~~~~~~
NC_049047.1_Dalbergia_chloroca  EPFFHAFPGGLEKIAINRIFLILPSRKEEREILFPFRRDQEIGSSRKKNRMLG*~~~~~~~~~~~~~~~~~~~~~~~~~~~~~~
```

```
NC_049050.1_Dalbergia_vietname   EPFFHAFPGGLEKIAINRIFLILPSRKEERERILFPFRRDQEIGSSRKKNRMLG*~~~~~~~~~~~~~~~~~~~~~~~~~~~~~
```
