# Supplementary material for: Comparative Analysis of Chloroplast Genomes of Dalbergia Species for Identification and Phylogenetic Analysis
Source: Plants (Basel). 2022 Apr 20;11(9):1109. doi: 10.3390/plants11091109 (PMC9104903; doi:10.3390/plants11091109)
Supplement: Supplementary file 1 [file plants-11-01109-s001.zip › Supp Table S1 - NGS reads and coverage.pdf]

Supp Table S1. Number of reads obtained in NGS and coverage of the five *Dalbergia* cp genomes.

Species	GenBank accession	No. of clean, paired-end reads	Assembly reads	Mean coverage (X)
Dalbergia hancei	OM328090	16,940,444	231,866	210.56
Dalbergia candenatensis	OM328093	16,579,278	906,938	829.73
Dalbergia assamica	OM328089	17,229,196	167,863	154.29
Dalbergia millettii	OM328092	19,675,290	324,230	296.26
Dalbergia benthamii	OM328091	19,290,536	208,723	189.36
