# Supplementary material for: Comparative Analysis of Chloroplast Genomes of Dalbergia Species for Identification and Phylogenetic Analysis
Source: Plants (Basel). 2022 Apr 20;11(9):1109. doi: 10.3390/plants11091109 (PMC9104903; doi:10.3390/plants11091109)
Supplement: Supplementary file 1 [file plants-11-01109-s001.zip › Supp Table S2 - SSR density.pdf]

Supp Table S2. Density of simple sequence repeat (SSR) of the five *Dalbergia* cp genomes.

Species	Regions	Length (bp)	# of SSR	SSR Density (# per bp)
Dalbergia hancei	LSC	85539	144	0.001683443
	IR	25671	6	0.000233727
	SSC	18978	37	0.001949626
Dalbergia millettii	LSC	85391	132	0.00154583
	IR	25723	6	0.000233254
	SSC	19070	35	0.001835343
Dalbergia benthamii	LSC	85767	145	0.001690627
	IR	25722	5	0.000194386
	SSC	19427	36	0.001853091
Dalbergia candenatensis	LSC	85253	144	0.00168909
	IR	25742	6	0.000233082
	SSC	19210	38	0.001978136
Dalbergia assamica	LSC	85290	146	0.001711807
	IR	25680	5	0.000194704
	SSC	19185	35	0.001824342
