# Supplementary figures and images for: Comparative Analysis of Chloroplast Genomes of Dalbergia Species for Identification and Phylogenetic Analysis
Source: Plants (Basel). 2022 Apr 20;11(9):1109. doi: 10.3390/plants11091109 (PMC9104903; doi:10.3390/plants11091109)

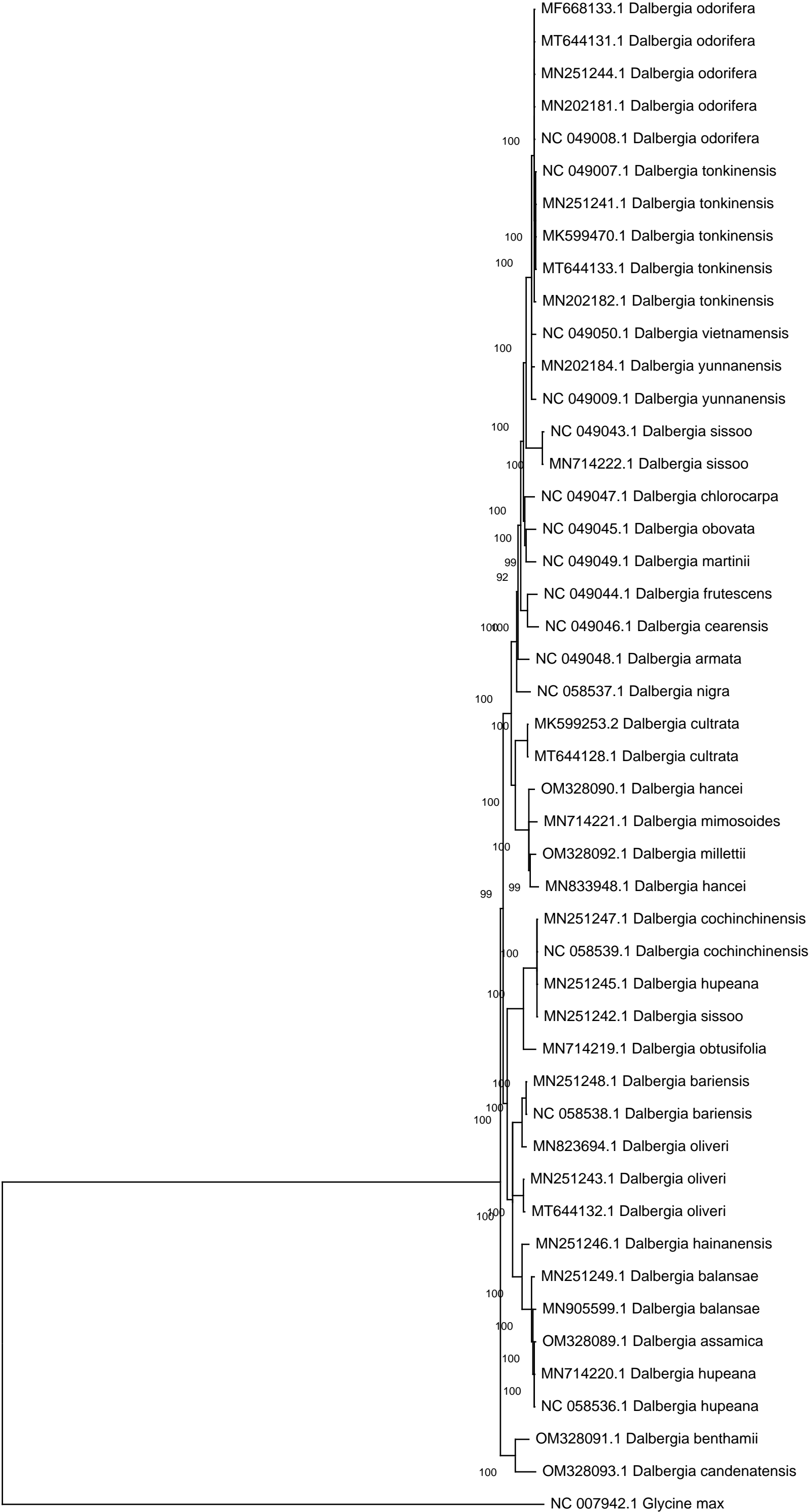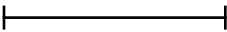

0.020

Supplement: Supplementary file 1 [file plants-11-01109-s001.zip › Supp Figure S2 - Original (un-condensed) maximum likelihood tree of 46 Dalbergia chloroplast genomes.pdf]

## Slide 1
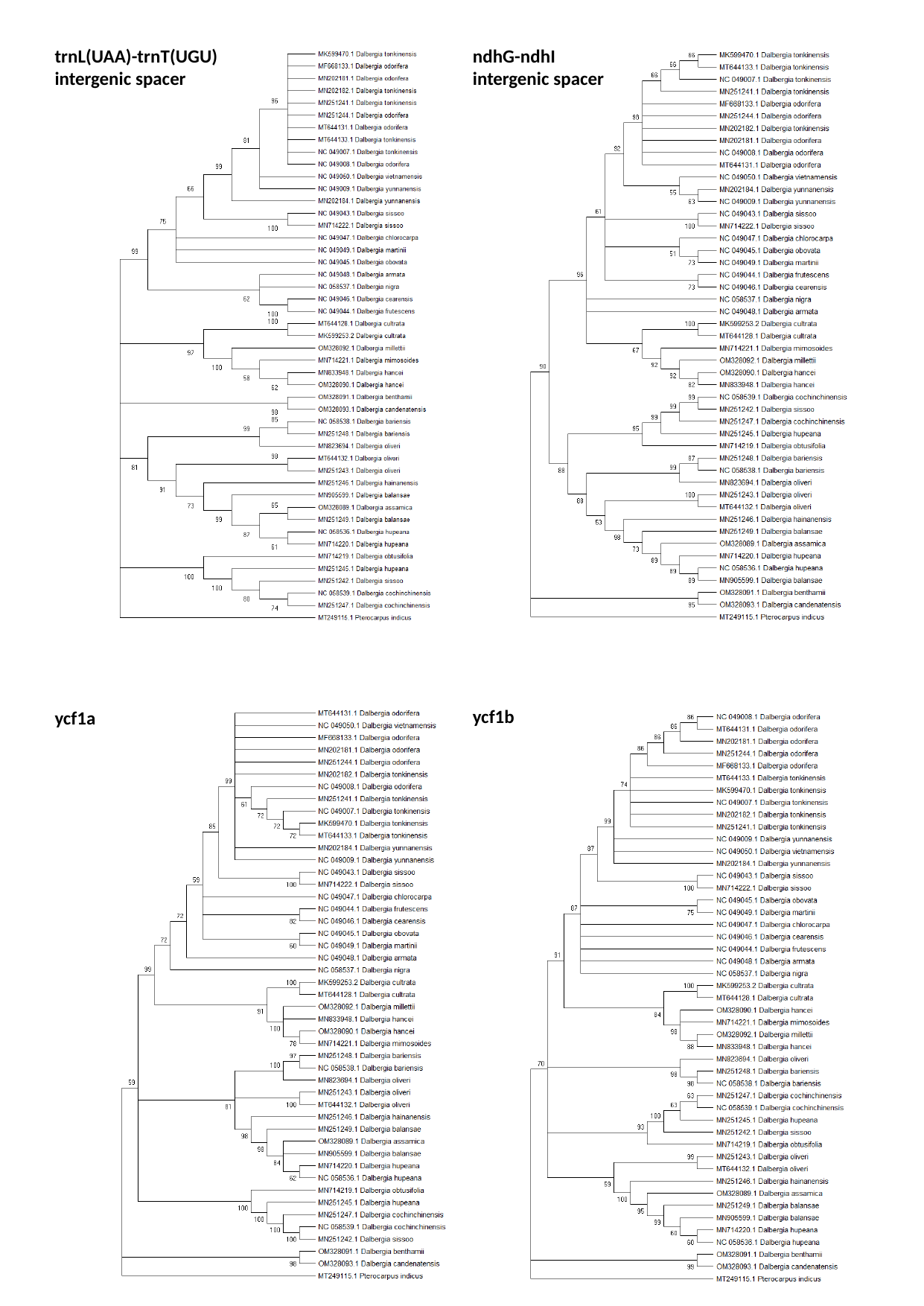

trnL(UAA)-trnT(UGU)
intergenic spacer
ndhG-ndhI
intergenic spacer
ycf1b
ycf1a

Supplement: Supplementary file 1 [file plants-11-01109-s001.zip › Supp Figure S3 - Hotspots NJ tree.pptx]

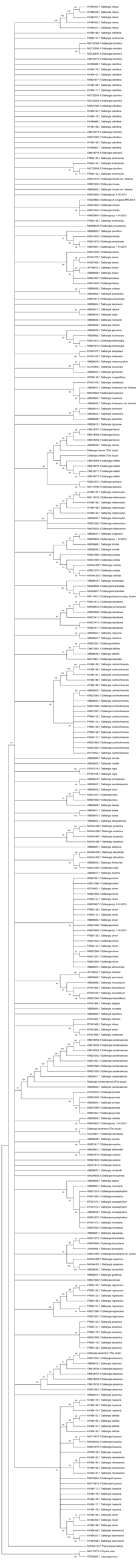

Supplement: Supplementary file 1 [file plants-11-01109-s001.zip › Supp Figure S4 - Neighbour joining tree of Dalbergia ITS2 (condensed at 50%, Pterocarpus, Glycine, Lotus as outgroup).pdf]
